# Human Tregs at the materno-fetal interface show site-specific adaptation reminiscent of tumor Tregs

**DOI:** 10.1172/jci.insight.137926

**Published:** 2020-09-17

**Authors:** Judith Wienke, Laura Brouwers, Leone M. van der Burg, Michal Mokry, Rianne C. Scholman, Peter G.J. Nikkels, Bas B. van Rijn, Femke van Wijk

**Affiliations:** 1 Center for Translational Immunology,; 2 Wilhelmina Children’s Hospital Birth Center,; 3 Regenerative Medicine Utrecht,; 4 Laboratory of Clinical Chemistry and Hematology, and; 5 Department of Pathology, Wilhelmina Children’s Hospital, University Medical Center Utrecht, Utrecht University, Netherlands.; 6 Obstetrics and Fetal Medicine, Erasmus MC University Medical Center Rotterdam, Rotterdam, Netherlands.

**Keywords:** Immunology, Reproductive Biology, Adaptive immunity, Obstetrics/gynecology, T cells

## Abstract

Tregs are crucial for maintaining maternal immunotolerance against the semiallogeneic fetus. We investigated the elusive transcriptional profile and functional adaptation of human uterine Tregs (uTregs) during pregnancy. Uterine biopsies, from placental bed (materno-fetal interface) and incision site (control) and blood were obtained from women with uncomplicated pregnancies undergoing cesarean section. Tregs and CD4^+^ non-Tregs were isolated for transcriptomic profiling by Cel-Seq2. Results were validated on protein and single cell levels by flow cytometry. Placental bed uTregs showed elevated expression of Treg signature markers, including FOXP3, CTLA-4, and TIGIT. Their transcriptional profile was indicative of late-stage effector Treg differentiation and chronic activation, with increased expression of immune checkpoints GITR, TNFR2, OX-40, and 4-1BB; genes associated with suppressive capacity (*HAVCR2*, *IL10*, *LAYN*, and *PDCD1*); and transcription factors *MAF*, *PRDM1*, *BATF*, and *VDR*. uTregs mirrored non-Treg Th1 polarization and tissue residency. The particular transcriptional signature of placental bed uTregs overlapped strongly with that of tumor-infiltrating Tregs and was remarkably pronounced at the placental bed compared with uterine control site. In conclusion, human uTregs acquire a differentiated effector Treg profile similar to tumor-infiltrating Tregs, specifically at the materno-fetal interface. This introduces the concept of site-specific transcriptional adaptation of Tregs within 1 organ.

## Abstract 2

Human regulatory T cells at the maternal-fetal interface show uterine site-specific functional adaptation with late-stage effector differentiation, chronic activation, Th1 polarization, and tumor-infiltrating, Treg-like features.

## Introduction

In the past decade, T cells have been identified in various human and murine nonlymphoid tissues (1, 2). These tissue-resident memory T cells (TRM) do not recirculate, serve as first-line responders to infections, and are characterized by expression of signature molecules such as CD69, which prevents their tissue egress (1, 3–8). TRM adapt to tissue environments by acquiring a specialized functional phenotype that depends on microenvironmental cues (9, 10). Also Tregs, critical gatekeepers of immune homeostasis (11), have been recently identified in murine and human tissues (12–16). Like TRM, Tregs can become resident and gain a polarized phenotype, with functional specialization depending on the tissue or organ, which is controlled on a transcriptional level (14–22). Although increasing evidence in mice supports functional adaptation of Tregs to nonlymphoid tissue environments (23), studies in humans are still scarce (14, 17). However, transcriptional adaptation of Tregs has gained special interest in the tumor environment, due to the important therapeutic implications (24). Tumor-infiltrating Tregs (TITR) display a unique and specialized transcriptional signature (25), associated with activation and functional specialization, including increased suppressive capacity (25–27). Tissue and tumor Tregs undergo differentiation reminiscent of effector Tregs, with potent suppressive capacity, and are characterized by expression of CD45RO and increased CD25, CTLA-4, and HLA-DR (28–31). Furthermore, effector Tregs (in tissues and tumors) express high levels of immune checkpoint molecules OX-40, 4-1BB, GITR, TIGIT, and ICOS and transcription factors such as BLIMP-1 (encoded by *PRDM1*) and BATF (19, 25–27, 31–34). Effector Tregs can mirror effector Th cell polarization by acquiring coexpression of FOXP3 with chemokine receptors and transcription factors associated with Th1 (CXCR3, T-bet), Th2 (GATA3, IRF4), or Th17 (RORγt, STAT3) differentiation (19, 35–38). This specific polarization is associated with an enhanced suppressive efficacy toward the matching T effector response (31, 36–45). Since most of these insights have been generated in mice, it is still largely unknown whether these principles also apply to human tissue Tregs.

As recently highlighted (46), one of the most interesting yet elusive tissue sites for Treg function in humans is the materno-fetal interface. Pregnancy is a mystifying biological process when viewed from an immunological perspective, posing a unique challenge to the maternal immune system (47, 48). While peripheral immunity against pathogens needs to remain intact, the semiallogeneic fetus and placenta, which may harbor foreign paternal antigens, need to be tolerated (49). The maternal immune response is therefore delicately balanced and requires tight regulation especially locally at the materno-fetal interface, which is underlined by the fact that human decidual T cells can recognize and actively respond to fetal cord blood cells (47–51). Maternal Tregs are consequently indispensable for successful embryo implantation and pregnancy outcome, and they contribute to materno-fetal tolerance on multiple levels (47, 52, 53). Depletion of murine maternal Tregs causes pregnancy loss due to immunological rejection of the fetus (53, 54). In humans, maternal Tregs are abundantly present in the gravid uterus (55–62), and normal human pregnancy is characterized by increased numbers of Tregs in the periphery and at the materno-fetal interface (56, 61, 63, 64). In patients with preeclampsia, a severe hypertensive pregnancy disorder, and patients with recurrent miscarriages, Treg numbers are reduced both at the materno-fetal interface and in the periphery (57, 65–70), implying that — also in humans — local presence of Tregs in the pregnant uterus is required for successful pregnancy outcome.

Previous studies investigating the maternal, uterine immune system in humans have been limited by the practical challenge of acquiring biopsy material of the uterine wall and have made use of the thin superficial decidual layer attached to the delivered placenta, which is heavily contaminated by fetal immune cells and may not be representative of the maternal Treg status during pregnancy. Moreover, the functional and transcriptomic profile of human uterine Tregs (uTregs) from the materno-fetal interface and its relation to Tregs from other human tissues remain to be elucidated. Here, we investigated functional adaptation and specialization of highly purified human, exclusively maternal, resident uTregs in myometrial biopsies from the materno-fetal interface. We performed transcriptomic profiling and functional in vitro assays, as well as flow cytometry, to study their phenotypic heterogeneity on protein level in single cell resolution. To identify tissue site–specific functional adaptation, we compared these uTregs with uTregs from a distant uterine control site and maternal peripheral bTregs, in addition to tissue- and site-matched resident CD4^+^ non-Tregs. Last, we observed the specific profile of functional adaptation of uTregs compared with known Treg signatures from other human and murine tissue sites, including tumors.

## Results

### uTregs are bona fide suppressive Tregs.

**Figure 1:**
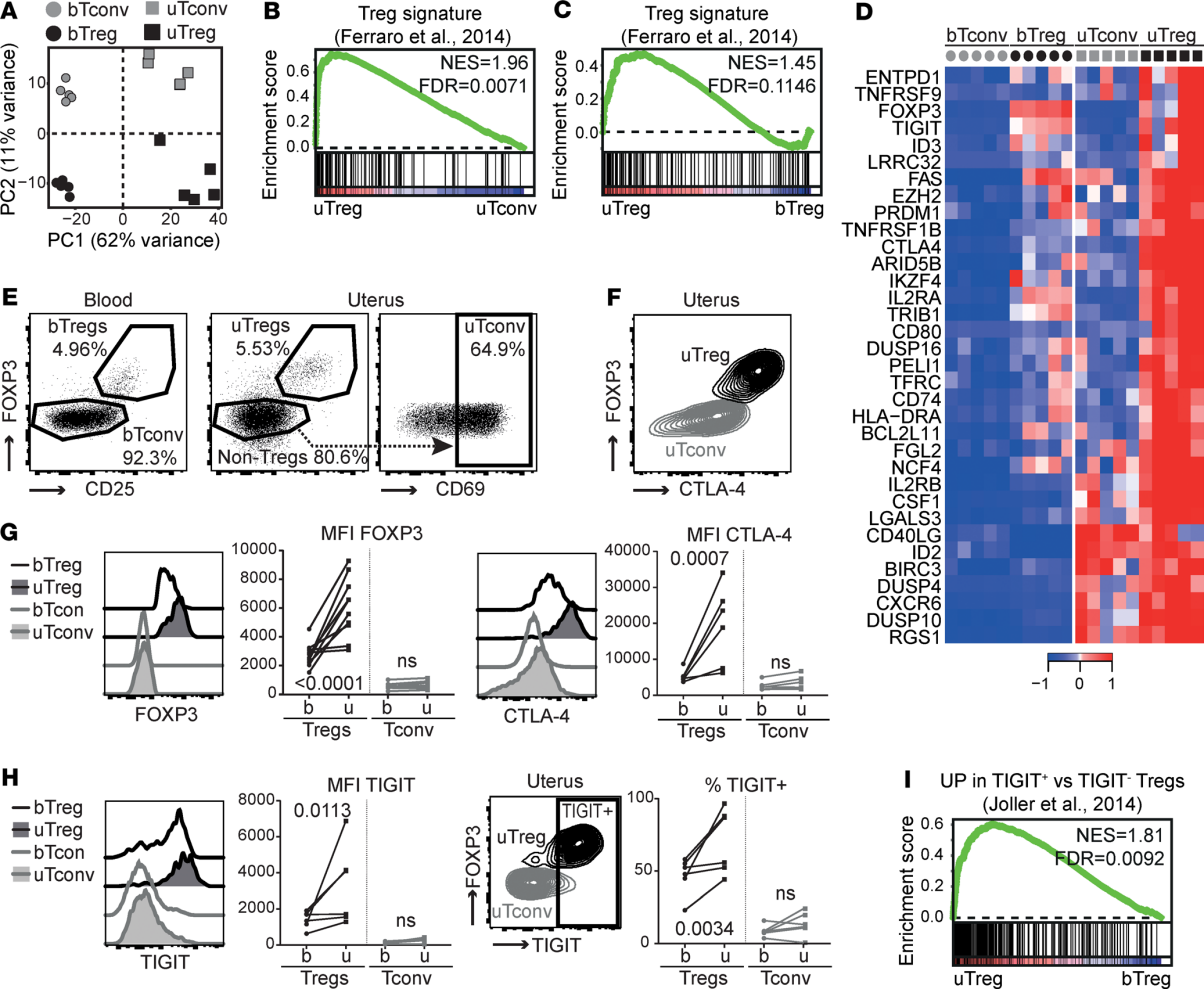
Tregs at the materno-fetal interface are bona fide Tregs. (**A**) Principal component analysis of bTregs, bTconv, uTregs, and uTconv (all *n* = 5). (**B** and **C**) GSEA with published Treg signature gene set (71) comparing uTreg and uTconv (**B**) and uTreg and bTreg (**C**) (all *n* = 5)**.** NES, normalized enrichment score; FDR, FDR adjusted *P* value. (**D**) Heatmap of genes in leading edge of GSEA comparing enrichment of published Treg signature genes in uTregs and bTregs. Expression values were mean centered and scaled per gene. (**E**) Representative gating strategy of bTregs, uTregs, and uTconv out of 5 experiments. (**F**) Representative expression of CTLA-4 in uTregs out of 6 experiments. (**G**) Ex vivo protein expression of core Treg molecules FOXP3 (*n* = 10) and CTLA4 (*n* = 6) measured by flow cytometry. (**H**) Ex vivo protein expression of Treg signature molecule TIGIT (*n* = 6) measured by flow cytometry. (**G** and **H**) Multiplicity adjusted *P* value of 2-way ANOVA with Tukey’s post hoc test. (**I**) GSEA of TIGIT^+^ Treg signature (*n* = 5) (72).

**Figure 2:**
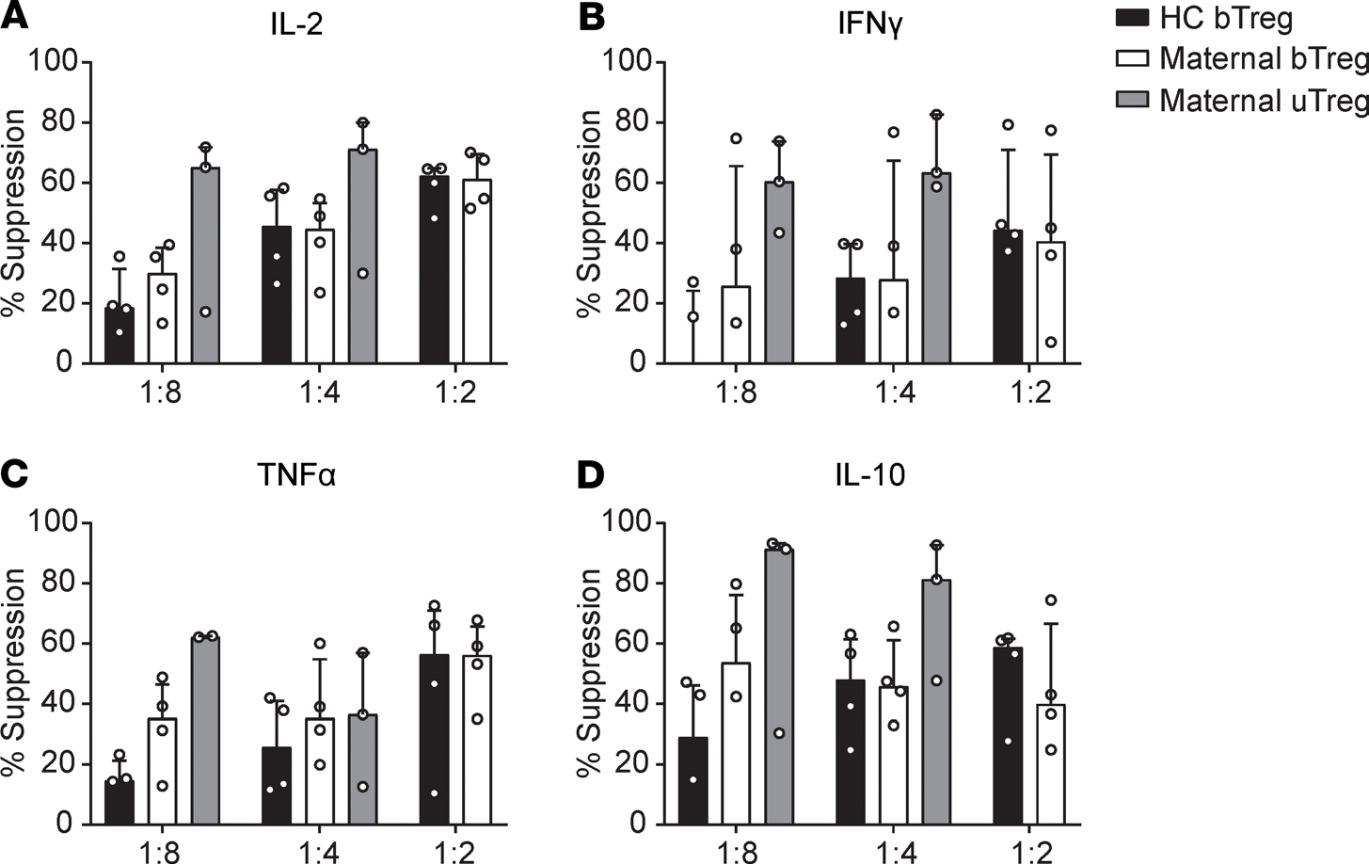
Tregs at the materno-fetal interface are bona fide Tregs with suppressive capacity. (**A–D**) Suppression assay assessing cytokine production of IL-2 (**A**), IFN-γ (**B**), TNF-α (**C**), and IL-10 (**D**) by anti-CD3–stimulated (or unstimulated) healthy CD4^+^ T cells in the supernatant by multiplex immunoassay after 4 days of coculture with healthy donor bTregs, maternal bTregs, or uTregs at a 1:8, 1:4, and 1:2 ratios. Data represent median with interquartile range. *n* = 4 donors, but not every condition could be measured for each donor due to limited availability of material. Therefore, some conditions contain data from 3 donors.

The frequency of CD25^hi^FOXP3^+^ Tregs within the CD4^+^ T cell population was similar between blood and uterine tissue and ranged from 2.5% to 13.5% (Supplemental Figure 1A; supplemental material available online with this article; https://doi.org/10.1172/jci.insight.137926DS1). For transcriptional profiling, the CD3^+^CD4^+^CD25^hi^CD127^–^ population (Tregs) and CD3^+^CD4^+^CD25^–^CD45RA^–^ memory T cells (Tconv) were FACS sorted from peripheral blood and myometrial biopsies from 5 women with uncomplicated pregnancies undergoing cesarean section. In myometrium, Tconv were selected for CD69 positivity. The sorting strategy is shown in Supplemental Figure 1B. Confirming the maternal origin of the sorted cells, the female-specific gene *XIST* was highly expressed in all samples, whereas transcripts of the male-specific gene *SRY* were undetectable in all samples, including pregnancies with male offspring (Supplemental Figure 1C). Principal component analysis (PCA) of transcriptomic profiles showed that uTregs from the materno-fetal interface are clearly distinct from blood-derived Tregs (bTregs), and that also uterine T conv (uTconv) and blood-derived Tconv (bTconv) clearly cluster apart (Figure 1A). Notably, PC1, mounting the difference between the cell sources, accounted for > 60% of the variance, whereas PC2, explaining variance between Treg and Tconv populations, accounted for only 11% of the variance. To assess whether the sorted population of uTregs consisted of bona fide Tregs, we analyzed enrichment of a published core Treg gene signature (71) in uTregs compared with uTconv and bTregs by gene set enrichment analysis (GSEA). Expression of Treg core signature genes was not only enriched compared with uTconv, but, remarkably, also more pronounced in uTregs than in bTregs, indicating that uTregs are bona fide Tregs with enhanced expression of Treg core signature genes (Figure 1, B and C). Indeed, expression of many of the published Treg markers (71) was higher in uTreg than bTreg (Figure 1D). Higher expression of the Treg-identifying molecules FOXP3 and CTLA-4 in uTregs than bTregs was confirmed on protein level (Figure 1, E–G). Also TIGIT, a key checkpoint molecule associated with specialized suppressive function (72), was highly expressed in uTregs, with the majority of uTregs being positive for TIGIT (Figure 1H). Consistently, GSEA showed significant enrichment of a previously identified TIGIT^+^ Treg signature (Figure 1I) (72). Suppression assays, although technically challenging due to low cell numbers, confirmed the suppressive potential of uTregs on proliferation and cytokine production of healthy donor peripheral blood–derived CD4^+^ T cells (Figure 2 and Supplemental Figure 1, D and E). Two of 4 uTreg donors showed particularly high suppressive capacity of uTregs on cytokine production of IL-2, IL-10, IFN-γ, and TNF-α, already at a 1:8 (Treg/Tconv) ratio, compared with bTregs. These results confirm that the sorted uTregs are bona fide functional Tregs, with enhanced expression of Treg signature genes.

### The uTreg signature indicates an activated and effector Treg profile.

**Figure 3:**
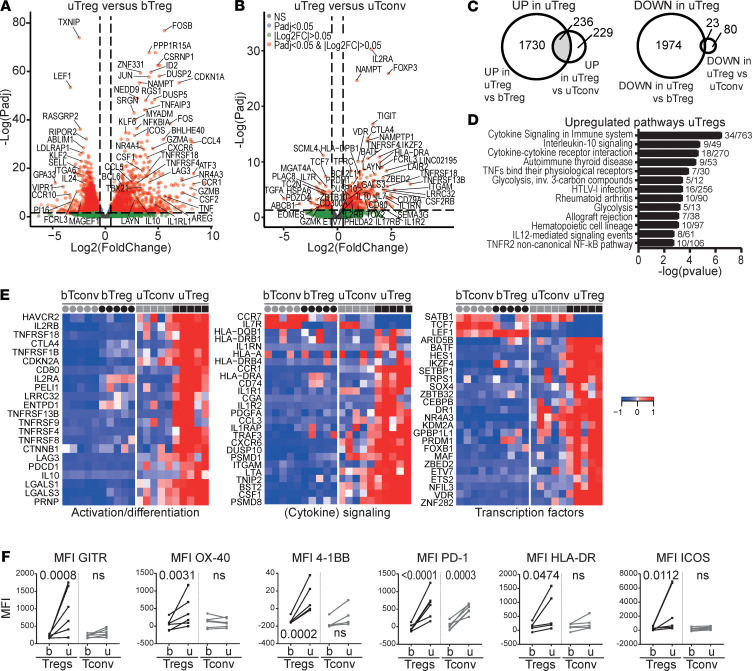
The uTreg core signature. (**A** and **B**) Volcano plot of differential gene expression between uTregs and bTregs (**A**) or uTregs and uTconv (**B**) (all *n* = 5). (**C**) Venn diagrams yielding genes specifically upregulated (*P*adj < 0.05 and log_2_FC > 0.5, left panel) or downregulated (*P*adj < 0.05 and log_2_FC < –0.5, right panel) in uTreg compared with bTreg and uTconv. (**D**) Pathway analysis (ToppGene pathways) of 236 genes specifically upregulated in uTregs. *P* < 0.05 after Bonferroni’s correction were considered significant. (**E**) Heatmaps showing gene expression of genes in top 5 pathways and selected downregulated genes in the uTreg core signature, related to Treg activation or effector differentiation (left panel), (cytokine) signaling (middle panel; including downregulated CCR7 and IL7R), and transcription factors (right panel). Expression values were mean centered and scaled per gene. (**F**) Protein expression of GITR (*TNFRSF18*), OX-40 (*TNFRSF4*), 4-1BB (*TNFRSF9*), PD-1 (*PDCD1*), HLA-DR, and ICOS. uTregs were gated as CD3^+^CD4^+^CD25^hi^FOXP3^+^ cells. Multiplicity adjusted *P* value of 2-way ANOVA with Tukey’s post hoc test. *n* = 6 each.

To investigate the functional adaptation of uTregs to the specific environment of the materno-fetal interface, we determined both their functional differentiation and Th polarization, both of which may be influenced by the tissue environment (12, 13, 20, 33, 73–75). To identify the uTreg-specific transcriptional signature, we assessed their differential gene expression with both bTregs and uTconv. Many genes were differentially expressed between uTregs and bTregs (Figure 3A), with significant upregulation of 1966 genes and downregulation of 1997 genes in uTregs (adjusted *P* value [*P*adj] < 0.05 and |log_2_FC| > 0.5). To isolate the uTreg-specific signature, we also compared gene expression between uTreg and uTconv, yielding 465 upregulated genes — including the Treg-identifying genes *FOXP3*, *IL2RA*, *CTLA4*, *TIGIT*, and *IKZF2* — and 103 downregulated genes in uTregs (*P*adj < 0.05 and |log_2_FC| > 0.5; Figure 3B); 236 genes were specifically upregulated (225 after removal of duplicate genes) and 23 genes specifically downregulated in uTregs compared with both bTregs and uTconv (Figure 3C, Supplemental Table 4, and Supplemental Table 5). Among the downregulated genes were *ITGA6*, *IL7R*, *CCR7*, *TTC39C*, *PLAC8*, *ATF7IP2*, *ABLIM1*, *MGAT4A*, *PRKCB*, and *GIMAPs*, as well as transcription factors *TCF7*, *LEF1*, and *SATB1*, indicating late-stage differentiation of Tregs (76, 77). The 225 upregulated genes were involved in cytokine signaling, TNF receptor signaling, and glycolysis (Figure 3D). Selected genes from the top 5 pathways included those related to Treg activation and effector differentiation, such as immune checkpoints of the TNF receptor superfamily (*TNFRSF13B* [TACI], *TNFRSF18* [GITR], *TNFRSF1B* [TNFR2], *TNFRSF4* [OX-40], *TNFRSF8* [CD30], *TNFRSF9* [4-1BB]) and *HLA-DR*, *CD80*, and *LRRC32* (GARP). Furthermore, genes associated with suppressive capacity (*CTLA4*, *ENTPD1*, *HAVCR2*, *IL10*, *IL2RA*, *LAG3*, *LAYN*, *LGALS1*, *PDCD1*, and *TOX2*) were highly expressed in uTregs (Figure 3E) (19, 31), and cytokine receptors of the IL-1 and IL-2 family (*IL1R1*, *IL1R2*, *IL1RAP*, *IL1RN*, *IL2RA*, and *IL2RB*) and specific chemokine receptors (*CCR1*, *CXCR6*) showed increased and specific expression in uTregs (Figure 3E). Transcription factors specifically upregulated in uTregs included *BATF*, *CEBPB*, *ETS2*, *ETV7*, *HES1*, *IKZF4*, *MAF*, *NFIL3*, *PRDM1*, *VDR*, and *ZBTB32* among others (Figure 3E). This transcriptomic profile, and especially high expression of *BATF*, *PRDM1*, and immune checkpoint molecules, reflects previously identified crucial signatures of effector Treg differentiation and function, especially in tissues (29, 32, 33, 78–80). We confirmed upregulation of immune checkpoints associated with effector Treg differentiation/chronic stimulation GITR, OX-40, 4-1BB, PD-1, HLA-DR, and ICOS in uTregs, even compared with uTconv, on the protein level (Figure 3F). Since increased expression of many of these genes pointed toward an activated phenotype, we confirmed this by demonstrating significant enrichment of published gene sets of in vitro–activated Tregs in uTregs (Supplemental Figure 2 and Supplemental Table 3) (81–85). Taken together, uTregs at the materno-fetal interface have a highly differentiated transcriptional signature suggestive of a specialized function with high suppressive capacity and high responsiveness to environmental cues, which is reflective of late-stage effector differentiation and chronic activation.

### uTregs have a tissue-resident phenotype and share transcriptional specialization with uTconv.

**Figure 4:**
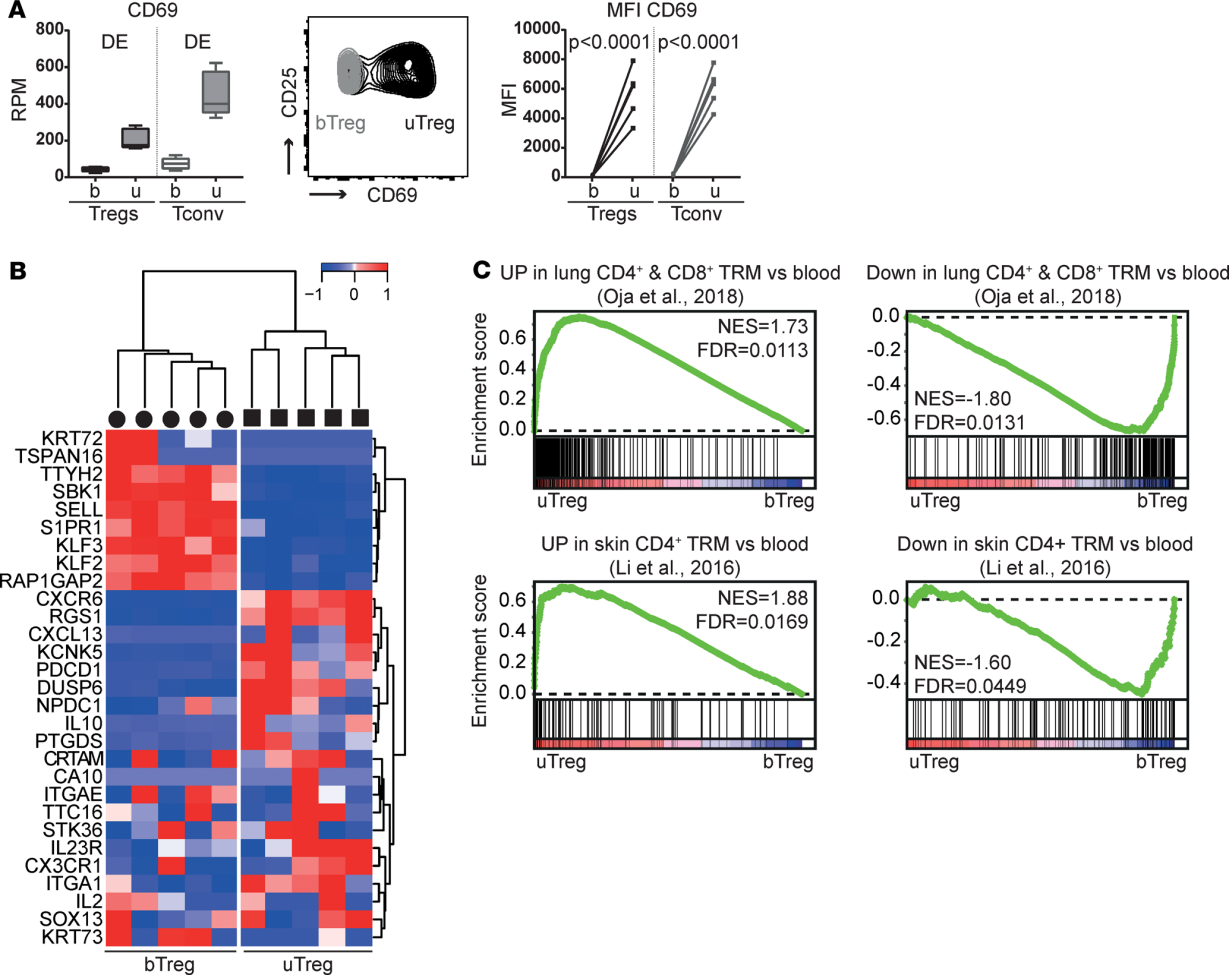
Tregs at the materno-fetal interface have a tissue-resident profile. (**A**) Gene and protein expression of CD69 in sorted T cell populations. DE, differentially expressed genes with *P*adj < 0.05. Box plots with median: box indicates 25th to 75th percentiles, whiskers indicate minimum and maximum values (*n* = 5). FACS data: representative plot of 5 experiments. uTregs were gated as CD3^+^CD4^+^CD25^hi^CD127^–^. MFI, median fluorescence intensity. Two-way ANOVA with Tukey’s post hoc test. (**B**) Heatmap with a published human core tissue–resident signature (4) in uTreg compared with bTreg. Expression values were mean centered and scaled per gene. (**C**) GSEA with published genes identifying human lung CD4^+^ and CD8^+^ TRM compared with blood memory cells; ref. 86) and genes upregulated/downregulated in skin CD4^+^ TRM compared with blood CD4^+^ T cells (14), in uTregs versus bTregs. NES, normalized enrichment score; FDR, FDR adjusted *P* value.

To examine whether uTregs at the materno-fetal interface represent a resident population or rather transiently infiltrating cells, we assessed the expression of tissue residency–related markers and gene signatures. uTregs had a significantly higher gene and protein expression of key residency molecule CD69 than bTregs and bTconv, similar to uTconv (Figure 4A). Expression analysis and GSEA with published human TRM signatures showed a pattern of upregulated and downregulated genes as previously described for human TRM in general (Figure 4B) and specifically in CD4^+^ (and CD8^+^) TRM from lung and skin (Figure 4C) (4, 14, 86), confirming the tissue-resident profile in uTregs as compared with bTregs. Next, we identified the shared tissue-specific adaptation of uTregs and uTconv to the materno-fetal interface. A large proportion of upregulated and downregulated genes was shared between uTregs and uTconv compared with their counterparts from blood (Supplemental Figure 3A; 1032 genes up and 1348 down; *P*adj < 0.05 and |log_2_FC| > 0.5), which again suggests that the specific tissue environment at the materno-fetal interface accounts for a significant part of their adapted transcriptional profile. Shared upregulated genes were involved in cytokine signaling (Supplemental Figure 3B), highlighting the integration of a spectrum of microenvironmental cues, while shared downregulated genes were reflective of ribosomal processes involved in RNA translation (Supplemental Figure 3C). Taken together, uTregs have a TRM signature that reflects a shared adaptation to the tissue environment of the materno-fetal interface between uTregs and uTconv.

### uTregs mirror uTconv Th1 polarization with a predominance of T-bet^+^CXCR3^+^ Tregs.

**Figure 5:**
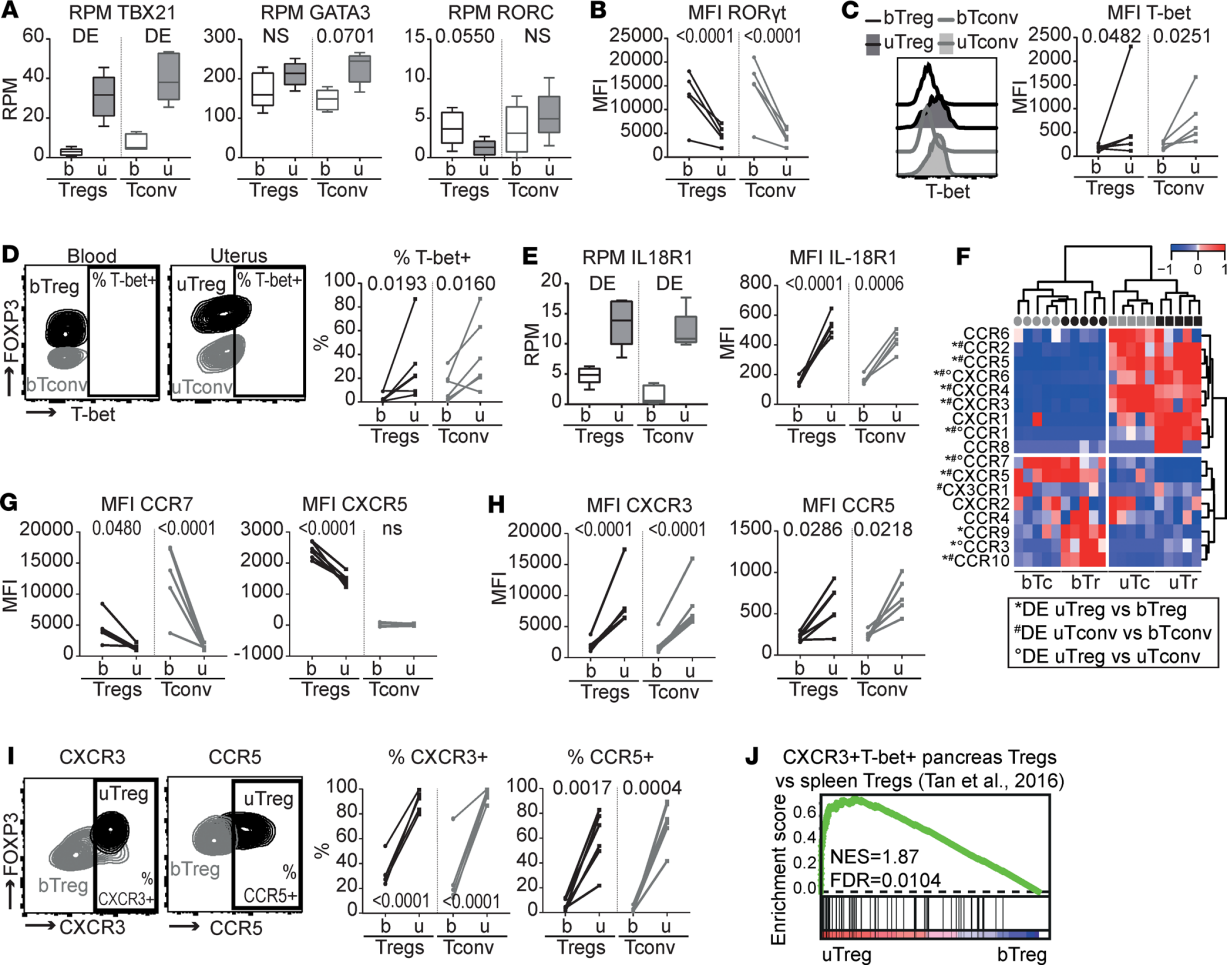
uTreg and uTconv polarization at the materno-fetal interface. (**A**) Gene expression of lineage-defining transcription factors *TBX21* (T-bet), *GATA3* (GATA-3), and *RORC* (RORγt). *P* values from differential gene expression analysis. DE, differentially expressed *P*adj < 0.05. Box plots with median; box indicates 25th to 75th percentiles, and whiskers indicate minimum and maximum values (all *n* = 5). (**B–D**) Protein expression of RORγt (**B**) and T-bet (**C** and **D**). uTregs were gated as CD3^+^CD4^+^CD25^hi^FOXP3^+^ cells. MFI, median fluorescence intensity. Multiplicity adjusted *P* values of 2-way ANOVA with Tukey’s post hoc test.(*n* = 5) (**E**) Gene and protein expression of *IL18R1* (IL-18R1). Gene expression: box plots with median — box indicates 25th to 75th percentiles, and whiskers indicate minimum and maximum values (*n* = 5). DE, differentially expressed *P*adj < 0.05. Protein expression: uTregs were gated as CD3^+^CD4^+^CD25^hi^FOXP3^+^ cells. Multiplicity adjusted *P* values of 2-way ANOVA with Tukey’s post hoc test (*n* = 5). (**F**) Heatmap showing gene expression of chemokine receptors. Expression values were mean centered and scaled per gene. DE, differentially expressed *P*adj < 0.05. (**G–I**) Protein expression of chemokine receptors downregulated (**G**) and upregulated (**H** and **I**) in uTregs. uTregs were gated as CD3^+^CD4^+^CD25^hi^FOXP3^+^ cells. *P* values of 2-way ANOVA with Tukey’s post hoc test (*n* = 5). (**J**) GSEA with published gene set of CXCR3^+^T-bet^+^ Tregs from the pancreas of prediabetic mice (38), comparing uTregs and bTregs. NES, normalized enrichment score; FDR, FDR adjusted *P* value.

Effector Tregs can acquire different Th phenotypes with coexpression of FOXP3 and lineage-defining transcription factors T-bet (*TBX21*, Th1), GATA3 (Th2), and RORγt (*RORC*, Th17), as well as lineage-associated cytokine and chemokine receptors (35). We investigated whether uTregs and uTconv underwent a, possibly shared, Th polarization. uTregs showed significantly increased expression of Th1-related *TBX21* compared with bTreg, which mirrored the increased expression of *TBX21* in uTconv (Figure 5A). Th2-related *GATA3* and Th17-related *RORC* were not significantly differentially expressed between uTreg and bTreg (and uTconv and bTconv), although *RORC* showed a trend toward downregulation, which was confirmed on the protein level (Figure 5, A and B). Increased expression of T-bet was also confirmed on the protein level, with 6%–87% (median 22%) of uTregs showing positivity for T-bet (Figure 5, C and D). Also, the Th1-related cytokine receptor IL-18R1 was increased in both uTregs and uTconv compared with blood T cells on the gene and protein levels (Figure 5E). Investigation of chemokine receptor expression, related to both Th polarization and tissue-specific homing (87, 88), showed that chemokine receptors associated with naive Tregs and lymphoid tissue environments CCR7 and CXCR5 were downregulated in uTregs compared with bTregs, on the gene and protein levels (Figure 5, F and G). Chemokine receptors upregulated in uTregs included *CCR2*, *CCR5*, *CXCR3*, *CXCR4*, *CCR1*, and *CXCR6* (Figure 5, F and H), which largely mirrored expression by uTconv. *CCR1* and *CXCR6* were specifically upregulated in uTregs, both previously identified as part of the conserved murine tissue Treg signature (18). The Th1-associated CXCR3 (36, 89) and Th1/inflammation-associated CCR5 (89–92) had significantly higher gene and protein expression in uTregs and uTconv compared with their counterparts from blood (Figure 5, F and H). Although the variable percentage of T-bet^+^ Tregs suggests heterogeneity in uTreg subspecialization, virtually all uTregs (and uTconv) were positive for CXCR3 (84%–100%, median 93%), and the majority expressed CCR5 (22%–83%, median 62%) (Figure 5I). Consistent with these findings, a previously published gene signature of T-bet^+^CXCR3^+^ Tregs from the pancreas of prediabetic mice was highly enriched in uTregs compared with bTregs (Figure 5J) (38). In conclusion, uTregs at the materno-fetal interface show Th1 polarization mirroring uTconv, with high expression of Th1-related markers T-bet and CXCR3. Furthermore, uTregs express an array of chemokine receptors with which they can integrate a variety of locally produced signals. While some of these receptors are uTreg specific, others are shared with uTconv. uTreg and uTconv cells may therefore rely on both unique and shared cues to guide their migration to and retention at the uterine materno-fetal interface.

### The uTreg signature at the materno-fetal interface overlaps with TITR signatures.

**Figure 6:**
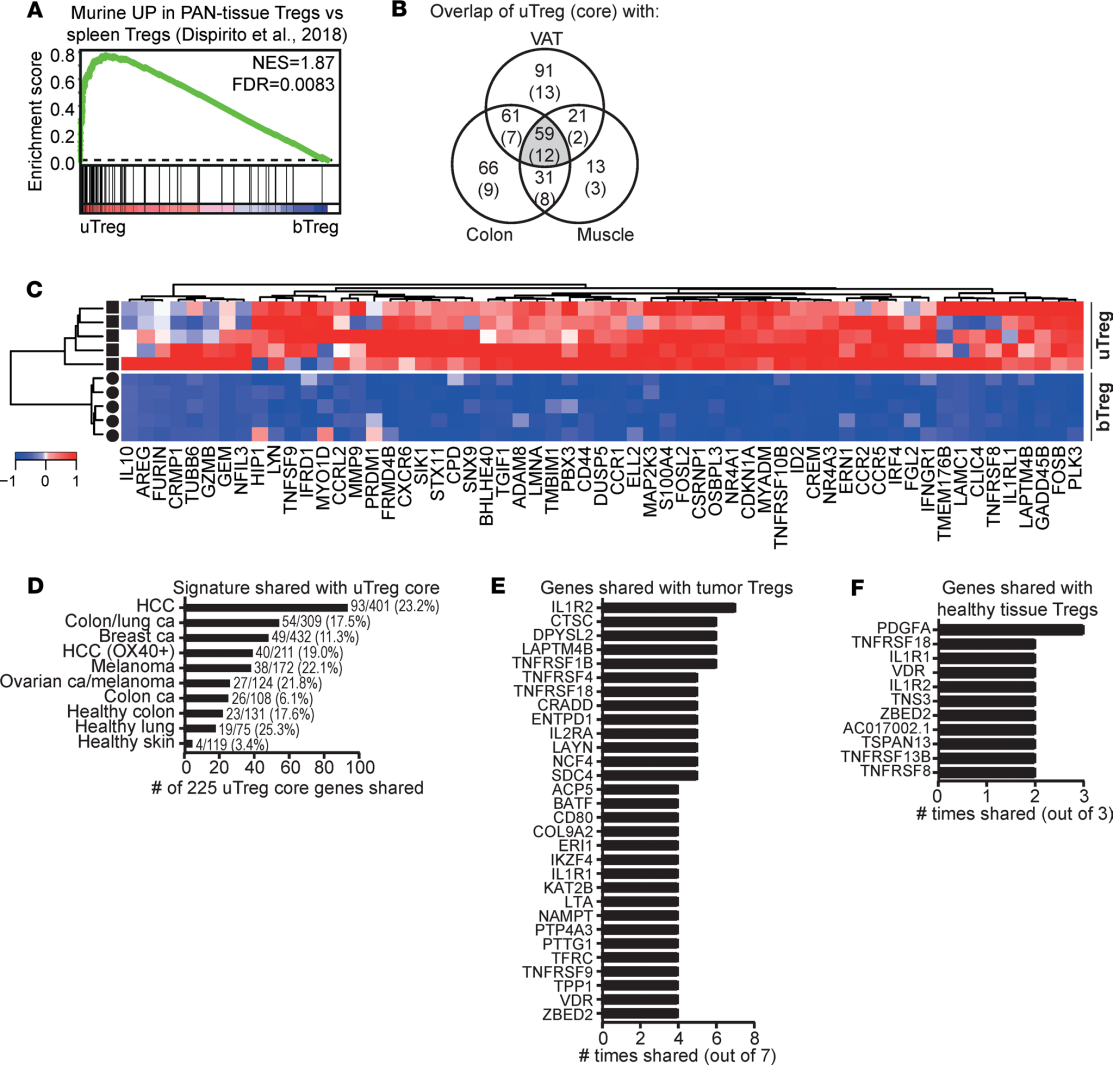
uTregs share their transcriptional signature with tissue- and tumor-infiltrating Tregs. (**A**) GSEA with a published murine PAN-tissue gene signature (18), comparing uTregs and bTregs. NES, normalized enrichment score; FDR, FDR adjusted *P* value. (**B**) Venn diagram showing the numbers of genes upregulated in uTregs compared with bTregs (*P*adj < 0.05) (and genes in the uTreg core signature in parentheses), which are represented in tissue-specific and tissue-shared published murine gene signatures (18). VAT, visceral adipose tissue. (**C**) Heatmap showing the expression of the 59 genes that were part of the murine PAN-tissue signature and upregulated in uTregs compared with bTregs (*P*adj < 0.05) (18). Expression values were mean centered and scaled per gene. (**D**) The number of genes shared between the uTreg core signature and published human TITR signatures or healthy tissue Treg signatures (14, 17, 25, 95–100). Numbers behind bars indicate the number of shared genes out of the total number of genes in the specific signature. (**E**) The genes that were most often shared between the uTreg core signature and human TITR signatures (shared in ≥ 4 of 7 signatures). (**F**) The genes that were most often shared between the uTreg core signature and human healthy tissue Treg signatures (shared in ≥ 2 of 3 signatures).

We questioned whether the highly differentiated uTregs from the materno-fetal interface would resemble Tregs from other human and murine tissue sites or would show a uniquely adapted profile. Well-studied murine tissue Treg populations include Tregs from visceral adipose tissue (VAT), muscle, and intestines (12, 16, 18, 73, 93). Each population displays a tissue-specific phenotype with expression of certain (a) transcription factors, (b) chemokine receptors, and (c) preference toward a Th lineage differentiation when compared with spleen Tregs (12, 13, 16, 18, 73). A murine PAN-tissue signature, shared by VAT, muscle, and intestinal Tregs, was also identified (18). GSEA in Figure 6A shows that the shared murine PAN-tissue Treg signature was also strongly enriched in uTregs, again highlighting its generalized expression in tissue Tregs, apparently even conserved across species. Overlaying significantly upregulated genes in uTreg (versus bTreg) with murine tissue–specific or murine tissue–shared Treg signatures (18), yielded a large amount of shared genes between uTregs and murine VAT–, colon–, and muscle–derived Tregs (Figure 6B, numbers in each field represent overlap of the specific field with significantly upregulated genes in uTreg). Fifty-nine genes were shared among all 3 murine tissues and uTregs, including *IL1RL1* (receptor for IL-33, ST2), *AREG*, *IL10*, *IRF4*, *GZMB*, *TNFRSF9*, *BHLHE40*, *NR4A1*, *NR4A3*, and *CCR2*, many of which have been described as crucial regulators for effector and/or tissue Treg function (Figure 6C) (12, 32, 40, 78–80, 94). Twelve of the 59 genes were even part of the uTreg-specific core signature as defined in Figure 3 (*CCR1*, *CXCR6*, *ELL2*, *FGL2*, *GEM*, *IL10*, *LAPTM4B*, *SNX9*, *TNFRSF8*, *NFIL3*, *NR4A3*, and *PRDM1*). This indicates that uTregs display features of tissue adaptation, which are highly conserved across tissues and species.

The investigation of human tissue–derived Tregs has proven challenging, and only limited data are available. To assess how uTregs compare with other human tissue Tregs, we analyzed enrichment of 3 previously published gene sets of significantly upregulated genes in healthy skin, colon, and lung Tregs compared with bTregs (Supplemental Table 3) (14, 17). All 3 signatures were significantly enriched in uTregs compared with bTregs, indicating that the tissue profile of uTregs shows similarities with human Tregs from various tissue sites (Supplemental Figure 4).

Human Tregs infiltrating the unique tissue-environment of tumors (TITR) have been studied more extensively. Comparison of genes significantly upregulated in uTregs versus bTregs with 7 recently published gene signatures of TITR infiltrating a variety of human tumors (Supplemental Table 3) (25, 95–100) yielded a remarkable overlap with each of the TITR signatures, with up to 65% of genes shared with uTregs (Supplemental Table 6). Of the 41 genes that were shared among ≥ 4 of the 7 TITR signatures (Supplemental Table 7), 31 were also part of the 225 genes in the uTreg core signature. Figure 6D shows the number of genes shared between the uTreg core signature, as well as each of the TITR signatures and healthy tissue–derived Treg signatures. Remarkably, 93 (41.3%) of the 225 core uTreg genes were overlapping with specifically upregulated genes from hepatocellular carcinoma–infiltrating (HCC-infiltrating) Tregs (98), 54 with the unique TITR signature identified by De Simone et al. (25), 49 with breast cancer TITR genes (95), and 40 with OX-40^+^ Treg from cirrhotic/tumor liver tissue (Figure 6D) (99). Importantly, the 225 uTreg core signature genes showed less overlap with healthy tissue–derived Treg–specific signatures from human healthy colon, lung, and skin. The genes that were most often shared between uTregs and TITR were *IL1R2* (7 of 7); *TNFRSF1B*, *CTSC*, *DPYSL2*, and *LAPTM4B* (6 of 7) and *TNFRSF4*, *TNFRSF18*, *LAYN*, *IL2RA*, *ENTPD1*, *NCF4*, *SDC4*, and *CRADD* (5 of 7) (Figure 6E), whereas — with healthy tissue-Treg signatures — *PDGFA* was most often shared (3 of 3) (Figure 6F).

**Figure 7:**
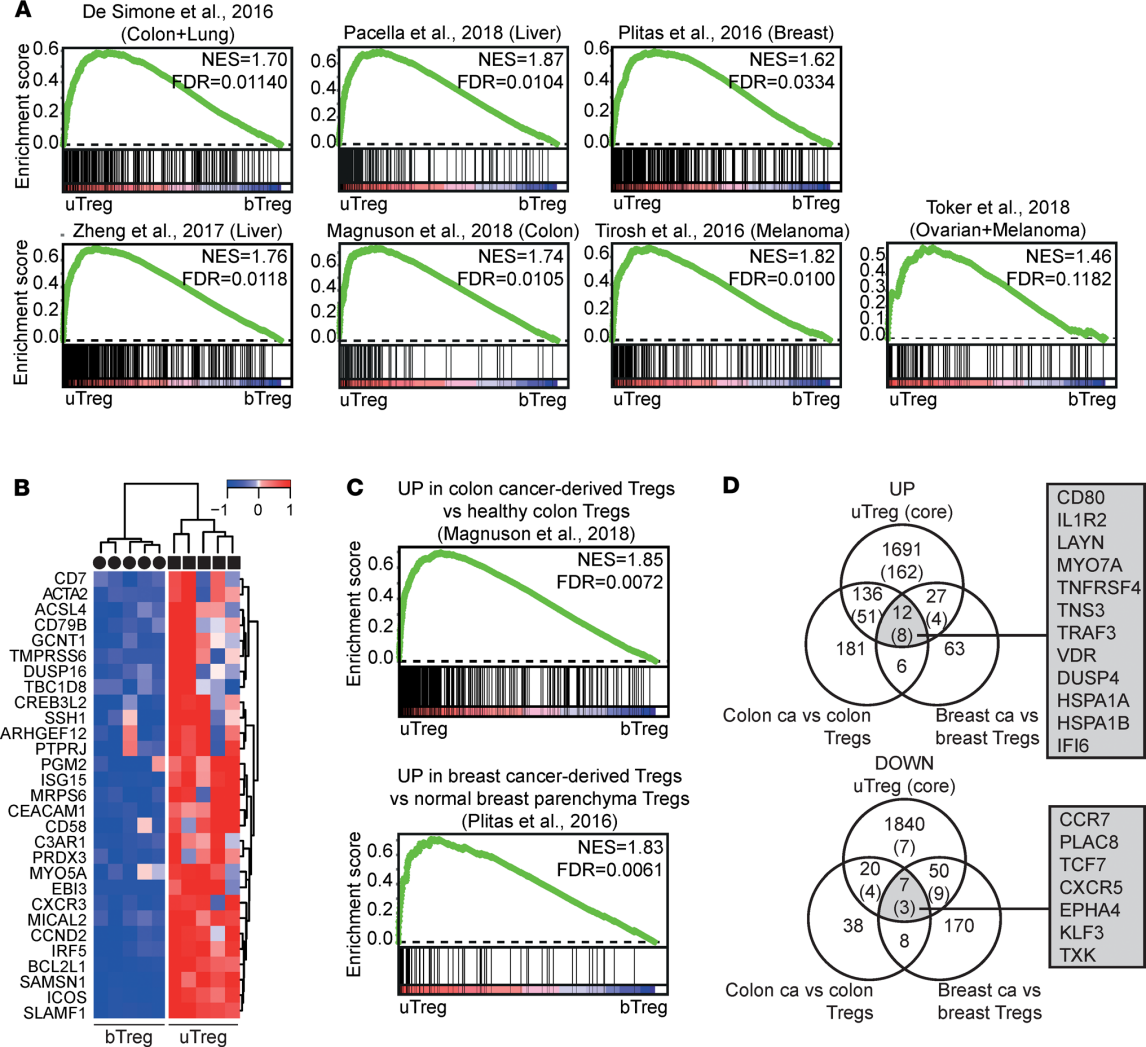
uTregs have a functional profile similar to tumor-infiltrating Tregs. (**A**) GSEA with published TITR-specific signatures in uTregs versus bTregs (25, 95–100). NES, normalized enrichment score; FDR, FDR adjusted *P* value. (**B**) Heatmap showing expression of genes in the leading edge of ≥ 3 of 7 GSEA from Figure 7A, which were not represented in the uTreg core signature. Expression values were mean centered and scaled per gene. (**C**) GSEA with published gene signatures specific to Tregs from tumor tissue compared with the healthy tissue counterpart in uTregs versus bTregs(26, 100). NES, normalized enrichment score; FDR, FDR adjusted *P* value. (**D**) Venn diagrams showing shared genes between uTregs and genes specifically upregulated in Tregs from tumor tissue compared with the healthy tissue counterpart (26, 100).

GSEA also showed that many of the nonoverlapping genes from the published TITR signatures were significantly enriched in uTregs compared with bTregs (Figure 7A). Genes in the leading edge of ≥ 3 of 7 tumor-specific GSEA that were highly expressed in uTregs compared with bTregs, but not part of the uTreg core (mostly because their high expression was shared with uTconv), are shown in Figure 7B. These included *CREB3L2* (6 of 7); *EBI3*, *GCNT1*, and *ICOS* (5 of 7); *ACTA2*, *ARHGEF12*, *BCL2L1*, *CCND2*, *PRDX3*, and *SLAMF1* (4 of 7); and *CXCR3*, *CD7*, *CAECAM1*, *CD79B*, and *MICAL2* (3 of 7), among others. Remarkably, genes specifically upregulated in breast cancer–infiltrating Tregs compared with Tregs from normal breast parenchyma or significantly upregulated in colon cancer Tregs compared with healthy colon Tregs showed a particularly high enrichment in uTregs, suggesting that uTregs are not just similar to Tregs from breast or colon tissue, but specifically to the highly differentiated/activated Tregs from the tumor environment (Figure 7C) (26, 100). By overlapping these cancer-versus-healthy tissue Treg signatures with significantly upregulated genes in uTregs (versus bTregs), we identified 12 cancer-specific genes expressed by uTregs (Figure 7D): *CD80*, *IL1R2*, *LAYN*, *MYO7A*, *TNFRSF4*, *TNS3*, *TRAF3*, *VDR*, *DUSP4*, *HSPA1A*, *HSPA1B*, and *IFI6*. The first 8 of these were also part of the uTreg-specific core signature, again highlighting the specificity c.q. importance of receptors *IL1R2*, *LAYN*, *TNFRSF4*, and *CD80* and transcription factor *VDR* for human Tregs in a tumor-like microenvironment. Furthermore, tumor-specific downregulated genes were shared with the uTreg core signature: *CCR7*, *PLAC8*, and *TCF7*. In conclusion, these results indicate that uTregs from the materno-fetal interface have a transcriptional core signature that is shared specifically with the specialized transcriptional profile of TITR.

### uTregs show site-specific adaptation within the uterus.

**Figure 8:**
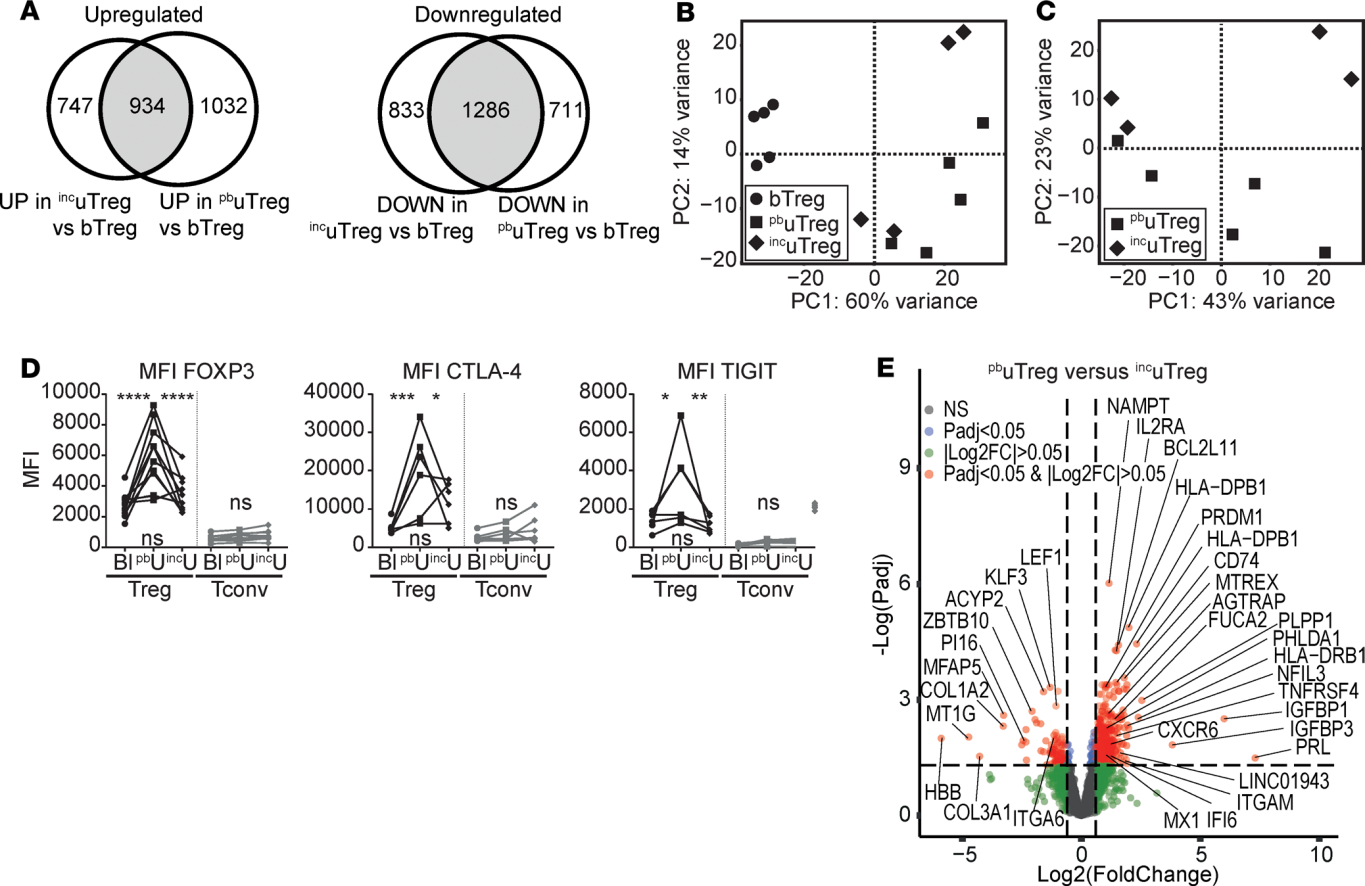
uTregs show site-specific adaptation to the materno-fetal interface. (**A**) Venn diagrams of genes upregulated (left panel) and downregulated (right panel) in both ^inc^uTregs and ^pb^uTregs compared with bTregs. (**B**) PCA of bTregs (*n* = 5), ^pb^uTregs (*n* = 5), and ^inc^uTregs (*n* = 4). (**C**) PCA of ^pb^uTregs (*n* = 5) and ^inc^uTregs (*n* = 4). (**D**) Protein expression of FOXP3, CTLA-4, and TIGIT. uTregs were gated as CD3^+^CD4^+^CD25^hi^FOXP3^+^ cells. Multiplicity adjusted *P* value of 2-way ANOVA with Tukey’s post hoc test. Left upper *P* value, blood versus placental bed; right upper *P* value, placental bed versus incision site; lower P value, blood versus incision site. MFI, median fluorescence intensity. (**E**) Volcano plot of differentially expressed genes between ^pb^uTregs and ^inc^uTregs. ****P < 0.0001, ***P < 0.001, **P < 0.01, *P < 0.05.

Next, we wondered whether uTregs would be merely adapted to the microenvironment in uterine tissue or specifically adapted to the tissue site at the maternal-interface. To investigate this site-specific adaption within 1 organ, we compared uTregs from the materno-fetal interface (i.e., placental bed; ^pb^uTregs) with uTregs from a distant uterine site (i.e., the incision site made during cesarean section; ^inc^uTregs). Confirmation of Treg identity and TRM signature for ^inc^uTregs is shown in Supplemental Figure 5, A–F. The differentially expressed genes between ^inc^uTregs and bTregs were similar to those between ^pb^uTregs and bTregs (Figure 8A). Furthermore, PCA showed that gene expression profiles of ^pb^uTregs and ^inc^uTregs were rather similar, compared with bTregs (Figure 8B). However, direct comparison of ^pb^uTregs and ^inc^uTregs revealed a substantial difference between the 2 populations (Figure 8C). First, protein expression of the core Treg transcription factor FOXP3 was lower in ^inc^uTregs than ^pb^uTregs, comparable with bTegs (Figure 8D). This was not due to ^inc^uTreg contamination with bTregs, since expression of CD69 was similar between ^pb^uTregs and ^inc^uTregs (Supplemental Figure 5D). Protein expression of other core Treg markers CTLA-4 and TIGIT was also lower in ^inc^uTregs than ^pb^uTregs (Figure 8D). This indicates that ^pb^uTregs, derived from the materno-fetal interface, have a more pronounced expression of Treg signature markers, suggesting enhanced activation/differentiation in comparison with their uterine counterparts from the incision site. Differential gene expression analysis revealed 558 upregulated and 125 downregulated genes in ^pb^uTregs versus ^inc^uTregs (Figure 8E).

**Figure 9:**
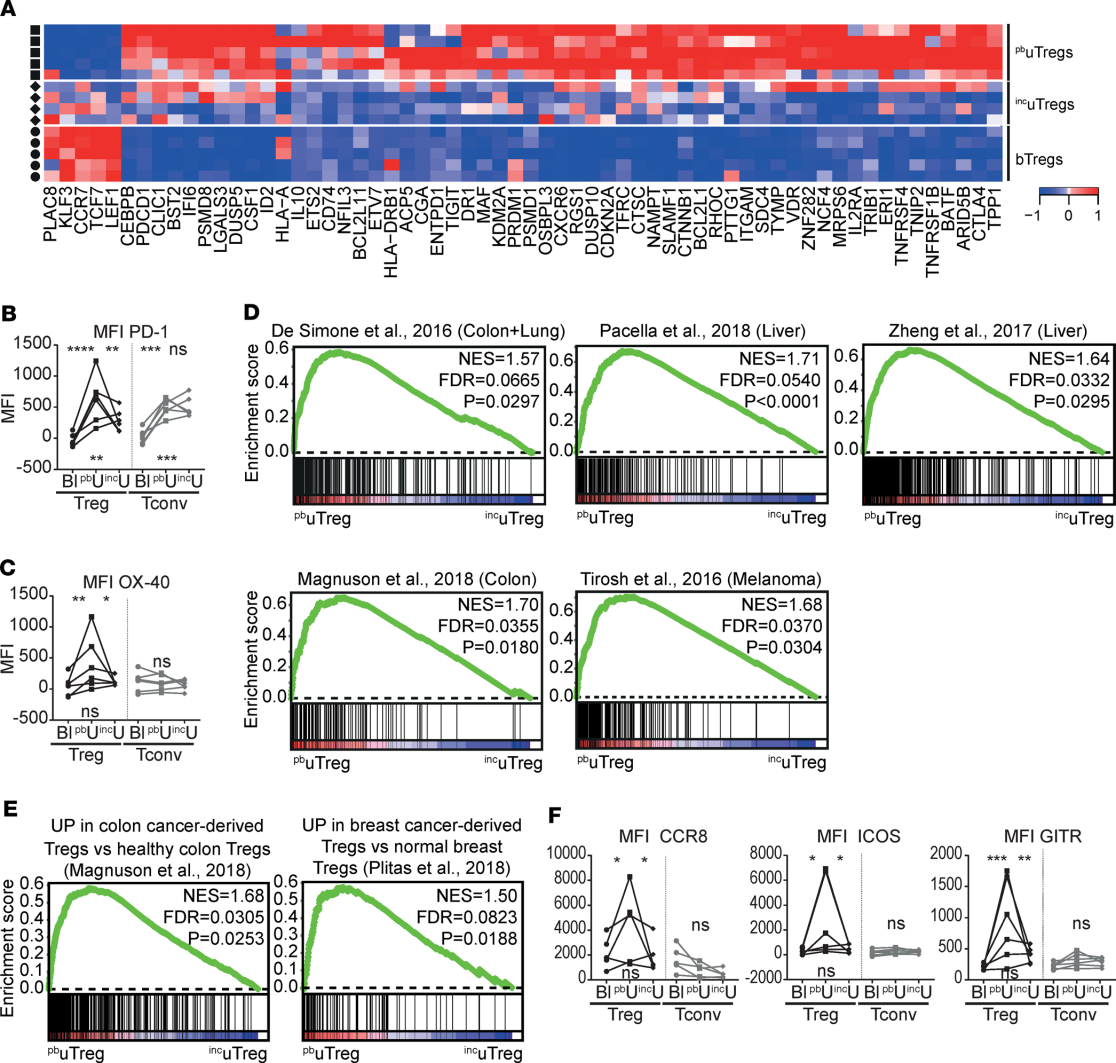
uTregs show site-specific adaptation to the materno-fetal interface. (**A**) Heatmap with previously highlighted genes in this manuscript that were differentially expressed between ^pb^uTregs and ^inc^uTregs. Expression values were mean centered and scaled per gene. (**B** and **C**) Protein expression of PD-1 (**B**) and OX-40 (**C**). uTregs were gated as CD3^+^CD4^+^CD25^hi^FOXP3^+^ cells. Multiplicity adjusted *P* value of 2-way ANOVA with Tukey’s post hoc test. Left upper *P* value, blood versus placental bed; right upper *P* value, placental bed versus incision site; lower *P* value, blood versus incision site. MFI, median fluorescence intensity (*n* = 6). (**D**) GSEA with published TITR-specific signatures in ^pb^uTregs versus ^inc^uTregs (25, 95–100). NES, normalized enrichment score; FDR, FDR adjusted *P* value. (**E**) GSEA with published gene signatures specific to Tregs from tumor tissue compared with the healthy tissue counterpart in ^pb^uTregs versus ^inc^uTregs (26, 100). (**F**) Protein expression of CCR8 (*n* = 5), ICOS (*n* = 6), and GITR (*n* = 6). uTregs were gated as CD3^+^CD4^+^CD25^hi^FOXP3^+^ cells. Multiplicity adjusted *P* value of 2-way ANOVA with Tukey’s post hoc test for protein. Left upper *P* value, blood versus placental bed; right upper *P* value, placental bed versus incision site; lower *P* value, blood versus incision site. MFI, median fluorescence intensity. ****P < 0.0001, ***P < 0.001, **P < 0.01, *P < 0.05.

The heatmap in Figure 9A shows a selection of previously highlighted genes in this manuscript that proved to be differentially expressed between ^pb^uTregs and ^inc^uTregs. These results suggest that Tregs cannot only adapt to the microenvironment within a certain tissue, but they will specifically adapt to the environmental cues at a certain tissue site. Pathway analysis showed that upregulated genes in ^pb^uTregs versus ^inc^uTregs were related to PD-1 signaling, cytokine signaling, TCR signaling, and Th cell differentiation (Supplemental Figure 5G). Indeed, PD-1 was expressed higher in ^pb^uTregs than ^inc^uTregs on gene and protein levels (Figure 9, A and B), and GSEA showed enrichment of a TCR-activated Treg signature in ^pb^uTregs compared with ^inc^uTregs (Supplemental Figure 5H). Furthermore, ^pb^uTreg-specific core genes associated with effector Treg differentiation including *TNFRSF4* (OX-40 protein, Figure 9C) and transcription factors *BATF*, *MAF*, *PRDM1*, and *VDR*, among others, were significantly expressed higher in ^pb^uTreg than in ^inc^uTregs (Figure 9A), again suggesting that ^pb^uTregs show more pronounced differentiation toward an effector Treg phenotype. Since ^pb^uTregs appeared to be especially differentiated at the materno-fetal interface, we assessed whether the TITR-like profile of ^pb^uTregs was also more pronounced than in ^inc^uTregs. Remarkably, 5 of 7 tested published TITR signatures were significantly enriched in ^pb^uTregs compared with ^inc^uTregs (*P* < 0.05; Figure 9D). More specifically, GSEA with signatures differentiating between TITR and their counterparts from a matched healthy tissue site showed significant enrichment in ^pb^uTregs compared with ^inc^uTregs (Figure 9E). *CCR8* and *ICOS*, which were present in 6 of 7 TITR signatures, as well as *TNFRSF18* (GITR), were expressed significantly higher in ^pb^uTregs than in ^inc^uTregs and bTregs on the protein level (Figure 9F). *CCR8* has been shown to be highly enriched in tumor Tregs and associated with a poor prognosis in several cancers (25, 26, 74). Thus, ^pb^uTregs at the materno-fetal interface specifically acquire a highly differentiated effector profile similar to TITR, which is more pronounced even compared with a uterine tissue site distant from the materno-fetal interface.

## Discussion

Here, we demonstrate for the first time to our knowledge that, in pregnancy, human uTregs have a highly differentiated transcriptional profile, which is specifically enriched at the materno-fetal interface and is reminiscent of the specialized profile of TITR. With these findings, we answer a long-standing question on how Tregs are functionally specialized at the materno-fetal interface to modulate local effector T cell responses, preventing an allo-reaction against the fetus. Moreover, we introduce the potentially novel concept of site-specific adaptation of Tregs within 1 organ or tissue. This again substantiates the notion that Tregs are capable of adapting their transcriptional program driven by microenvironmental cues (15–19).

We have demonstrated that uTregs at the materno-fetal interface display a highly activated and late-stage differentiated effector profile (32, 34, 76, 77, 79), with high BATF and PRDM1, low SATB1, increased expression of molecules associated with suppressive capacity, and abundant expression of TNFR superfamily members. Also, others have reported that nonlymphoid tissue Tregs display an activated phenotype compared with lymphoid organ and circulating Tregs (12, 20, 73). Both *BATF* and the TNFRSF/NF-κB signaling axis are crucial in the survival of Tregs and maintenance of a stable effector Treg phenotype, especially in tissues (15, 28, 32, 33, 78, 79, 101). It is now recognized that Tregs adapt to their tissue environments, with, on the one hand, common adaptations across many tissues — such as increased expression of IL10, IL1RL1 (encoding ST2, an IL-33 receptor subunit), AREG (encoding amphiregulin), CTLA4, TIGIT, BATF, and IRF4 and decreased expression of LEF1, TCF7 — but, on the other hand, importantly, tissue-specific signatures (12, 13, 15, 16, 18). These tissue-specific signatures counter the notion that tissue Tregs merely have a more activated effector or memory state. Rather, they have a specialized adapted program (93), likely matching the specific requirements of a certain tissue site (10, 13, 18, 102, 103).

To our knowledge, the concept of site-specific transcriptional adaptation of Tregs within 1 tissue or organ is novel, taking into account that tumors represent a completely altered tissue and not a different site within the same organ. We show that uTregs display features suggestive of a high responsiveness to microenvironmental cues, such as a range of TNF receptor superfamily members and chemokine receptors. With such a matrix of options to detect signals from the microenvironment, Tregs are likely able to adjust not only to the tissue or organ of their residence, but even to specific sites within that tissue, based on microenvironmental cues. Most likely, implantation of the placenta (i.e., the multitude of signals produced by myometrium-invading trophoblast; ref. 104) are the primary cues effectuating microenvironmental changes at the materno-fetal interface. Trophoblast attracts Tregs to the materno-fetal interface by production of hCG and CXCL16, the ligand for CXCR6.(105, 106). Moreover, in vitro coculture of HLA-G^+^ extravillous trophoblast with CD4^+^ T cells increased Treg numbers and FOXP3 expression level (107, 108), indicating that Tregs may also be locally induced or expanded by trophoblast. Thus, it is likely that signals produced by invading trophoblasts at the materno-fetal interface account for at least some of the site-specific transcriptional adaptations in uTregs.

The Th response at the materno-fetal interface was previously suggested to be skewed away from a proinflammatory Th1 response, to prevent a pathogenic allo-reaction against the fetus, resulting in a Th2-dominant response during the second trimester. However, during the third trimester, a proinflammatory Th1 response may be essential for initiation of labor (reviewed in ref. 48). In line with this, our findings indicate that the Th response in the uterus at term is dominated by Th1 polarization, although well controlled. uTregs at the materno-fetal interface appear to be specifically equipped to effectively suppress Th1 responses. Although we observed heterogeneity of T-bet protein expression in uTreg, CXCR3 expression was remarkably homogeneous, with 84%–100% of uTregs being CXCR3^+^. CXCR3-expressing (and T-bet– expressing) Tregs are especially adept to suppress Th1 responses (36, 38, 45, 89). Furthermore, the majority of uTregs expressed TIGIT, OX-40, and/or CCR5. Tregs expressing TIGIT preferentially inhibit Th1 and Th17 responses (72), a subpopulation of OX-40–expressing Tregs can differentiate into Th1-suppressing Tregs (109), and also CCR5 expression on Tregs has been associated with more effective suppression of Th1 responses (90). Thus, the necessary proinflammatory Th1 response at the materno-fetal interface at term appears to be controlled by specifically differentiated and Th1-polarized Tregs. So far, Th1-like Tregs have been described mainly in inflammatory environments, such as infections, autoimmune diseases, and transplantation reactions (45, 89, 110–112), whereas tissue-resident Tregs were mostly characterized as being Th2 skewed (VAT, muscle) (13, 18) or Th17 skewed (intestines) (73). DiSpirito et al., however, recently also identified a subset of T-bet–expressing Tregs in muscle and colon (18), indicating that they can also be present in steady-state tissues.

We are the first to our knowledge to study exclusively maternal, myometrial tissue–resident Tregs from the materno-fetal interface. Previous studies of uTregs had to resort to the use of more easily accessible decidua, due to the difficulty of acquiring human myometrium. Since decidual tissue is of fetal origin, it may not only be contaminated with fetal immune cells, but it also does not allow for studying the unique, and specifically maternal, uterine environment underlying the placenta, in which the complex process of spiral artery remodeling takes place. The only publications that we know of investigating FOXP3 expression in actual human placental bed biopsies demonstrated that the percentage of FOXP3^+^ T cells was significantly decreased in patients with preeclampsia, and FOXP3 mRNA expression was reduced in endometrial biopsies of infertile women, highlighting the importance of functional Tregs for a healthy pregnancy (57, 113). From human decidual data, it is known that the frequency of clonally expanded populations of effector Tregs is increased in decidua of third trimester cases compared with first trimester cases (114). Decidual Tregs display a more pronounced suppressive phenotype than in blood, with increased expression of FOXP3, CTLA-4, CD25, HLA-DR, ICOS, GITR, and OX-40, which recapitulates our findings (58, 59, 63, 115). Recently, 3 types of functional Tregs were identified at the human materno-fetal interface, of which the CD25^hi^FOXP3^+^ population matches the population investigated in this study (108). These Tregs effectively suppressed CD4^+^ and CD8^+^ T cell proliferation and IFN-γ and TNF-α production. Transcripts identified by qPCR array as specific for this subset were *IL2RA*, *FOXP3*, *TIGIT*, *ENTPD1*, *LRRC32*, *IL1RL1*, *BATF*, and *CCR8*, and increased expression of *CCR5*, *IL10*, and *GITR* compared with bTregs was also observed (108), which confirms our findings of an activated Treg phenotype at the materno-fetal interface. A study investigating chemokine receptor expression of CXCR3, CCR4, and CCR6 in decidual Tregs by flow cytometry showed that CCR6^–^CXCR3^+^ Th1 cells were increased and CCR6^+^CCR4^+^ Th17 cells were nearly absent, whereas CCR4^+^ Th2 frequencies were similar in blood and decidua (58), which is also in line with our findings. Taken together, this indicates that the here-identified activated phenotype of myometrial uTregs has overlapping characteristics with decidual Tregs.

We observed that uTregs from the materno-fetal interface display a peculiar differentiated effector phenotype similar to TITR, defined by high expression of *IL1R2*, *LAYN*, *CD80*, *VDR*, and *TNFRSF4*, among others, with specific enrichment of TITR signatures compared with Treg signatures from matched, unaffected tissue sites. This observation may be explained by recent insights on the similarity of the immune environment at the materno-fetal interface and tumors (48). Both the receptivity of the myometrium toward implantation of the blastocyst and invasiveness of the trophoblast show striking similarities with implantation of tumor metastases in healthy tissues (116, 117). Tumor cells can modulate their immune environment into an antiinflammatory milieu and can recruit and/or induce suppressor cells, among which are high numbers of Tregs (118, 119). Just as in tumors, a tolerogenic mode of antigen presentation with indirect allorecognition of low levels of antigens predominates at the materno-fetal interface (120). Furthermore, others reported striking similarities between early Treg responses to embryo and tumor implantation (54). These findings imply that the microenvironment at the materno-fetal interface may be a unique mammalian tissue site that, under challenged but physiological conditions, resembles a tumor microenvironment: an actively remodeling tissue site distinct from a steady-state tissue, with low-grade inflammation and newly infiltrating/invading cells. These dynamic characteristics may account for the unique transcriptional adaptation of Tregs.

Although we observed global changes in gene expression patterns in uTregs, flow cytometry revealed an expression gradient of many markers across the uTreg population, suggesting that uTregs consist of a heterogenic population with various stages of differentiation and possibly subspecialization. Single cell sequencing techniques and mass cytometry are indeed starting to reveal the heterogeneity of Treg populations in tissues and tumors (15, 27, 97, 98, 121–123).

A unique strength of our study is that we compared the transcriptomic profile of a highly specific and highly purified maternal Treg subset from myometrial biopsies, not only to their counterpart in blood, but also to a tissue-specific and site-specific Treg control population and matched Tconv population. We validated our key findings on the protein level in single cell resolution by flow cytometry. We only studied term pregnancies, due to the practical limitation of delivery of the infant and placenta. It would therefore be interesting to investigate term-dependent changes in uTreg profiles in future studies. Although protocols for tissue digestion may induce transcriptional changes (124), many of the uTreg-specific genes were previously found not to be affected by a tissue digestion protocol similar to but harsher than the one used here (17).

Our findings have important implications. TITR are currently under heavy investigation as targets in cancer immunotherapy. However, we demonstrate that signatures identified in TITR are not as unique as previously assumed and that they may be shared by Tregs with specialized functions in other human tissues that may still be unknown. On the other hand, our results may lead to new targets for cancer immunotherapy, since profiling of Tregs in a variety of tissues under physiological, but not necessarily steady-state, conditions may help to identify truly TITR-specific expression patterns. Moreover, increased understanding of immunoregulatory mechanisms at the materno-fetal interface during healthy pregnancy gives unique insights into human immunobiology of pregnancy and also aids to elucidate the pathological changes in Tregs in pregnancy disorders such as preeclampsia, fetal growth restriction, or recurrent miscarriage, as many studies have pointed toward a role for Treg defects or deficiency in these disorders (65–67, 105, 114, 125, 126). Last, functional adaptation of human Tregs to different tissues and specific tissue sites is still largely unexplored. The receptivity of Tregs to their environmental stimuli and subsequent subspecialization may be exploited for therapeutic purposes.

In conclusion, we have shown that human Tregs show functional adaptation with tumor-infiltrating–like features specifically at the materno-fetal interface, which introduces the concept of tissue site-specific transcriptional adaptation of human Tregs.

## Methods

### Participants and biopsies.

This study is part of the Spiral Artery Remodeling (SPAR) cohort study, which is an ongoing effort to investigate the adaptation of the uterus to placental development by obtaining site-specific uterine biopsy samples in women undergoing cesarean section. A detailed description of the study set-up and protocol was previously published (127). For this analysis, we included 20 women who delivered by elective cesarean section (i.e., without any contractions or other signs of labor such as rupture of membranes) after an uncomplicated pregnancy and without any major underlying pathology, *n* = 5 of which were included for transcriptomics of T cell populations, *n* = 4 for suppression assays, and *n* = 11 for flow cytometry. Baseline characteristics are provided in Supplemental Table 1. One tube of sodium-heparin blood was taken from each donor before cesarean section. After delivery of the neonate and placenta, the placental bed was manually located, and 2 biopsies of the central placental bed from the inner uterine myometrial wall were obtained as previously described (127). Additionally, biopsies were taken from the incision site when the placenta was not situated on this part of the uterine wall.

### Lymphocyte isolation.

Peripheral blood mononuclear cells (PBMC) were isolated from blood diluted 1:1 with basic medium (RPMI 1640 [Thermo Fisher Scientific] with penicillin/streptomycin [Thermo Fisher Scientific], L-glutamine [Thermo Fisher Scientific]), by ficoll-density centrifugation (at room temperature, 1100*g* for 20 minutes; GE Healthcare-Biosciences, AB). PBMC were washed in basic medium with 2% FCS (Biowest) and PBS or staining buffer consisting of cold PBS supplemented with 2% FCS and 0.1% sodium-azide (Severn Biotech Ltd.). The biopsy samples were collected in basic medium supplemented with 10% FCS and minced into pieces of 1 mm^3^ in PBS (Thermo Fisher Scientific). The biopsies were enzymatically digested with 1 mg/mL collagenase IV (MilliporeSigma) in medium for 60 minutes at 37°C in a tube shaker under constant agitation at 120 rpm. To dissolve the remaining biopsy pieces after digestion and remove any remaining lumps, the biopsies were pipetted up and down multiple times and poured over a 100 μm cell strainer (BD Falcon). Cells were subsequently washed in staining buffer and filtered through a 70 μm cell strainer, and they were prepared for flow cytometry or flow cytometry-assisted cell sorting.

### Flow cytometry.

For flow cytometric experiments without restimulation, PBMC and uterine cells were first incubated in fixable viability dye eFluor506 (eBioscience) in PBS (1:300) for 20 minutes at 4°C and washed in PBS. For surface staining, cells were incubated with the antibodies shown in Supplemental Table 2 for 20 minutes in staining buffer at 4°C and were subsequently washed in the same buffer. Cells were permeabilized with 1 part fixation/permeabilization concentrate and 3 parts fixation/permeabilization diluent (eBioscience) for 30 minutes at 4°C and subsequently incubated overnight with intracellular antibodies (Supplemental Table 2) in 10× diluted Permeabilization buffer (Perm; eBioscience) 4°C. The next day, cells were washed with Perm and measured on the LSR Fortessa (BD Biosciences). For intracellular cytokine measurement, PBMC and uterine cells were first incubated with surface staining, washed, and then restimulated with 20 ng/mL phorbol 12-myristate 13-acetate (PMA, MilliporeSigma) and 1 μg/mL ionomycin (MilliporeSigma) for 4 hours with addition of Monensin (Golgistop, BD Biosciences) during the last 3.5 hours at 37°C. Afterward, cells were stained with the viability dye, permeabilized, intracellularly stained, and measured as described above.

### Flow cytometry–assisted cell sorting.

Cells were incubated with surface antibodies (Supplemental Table 2) for 20 minutes in staining buffer at 4°C, washed in the same buffer, and filtered through a 50 μm cell strainer (Filcon, BD Biosciences). For suppression assays, cells of the CD3^+^CD4^+^CD25^+^CD127^–^ cell population (Tregs) and CD3^+^CD4^+^CD25^–^ cell population (Tconv) were directly sorted into tubes with 500 μL FCS on a FACSAria III (BD Biosciences). For RNA sequencing (RNA-seq), 2000 cells of the CD3^+^CD4^+^CD25^+^CD127^–^ cell population (Tregs) and CD3^+^CD4^+^CD25^–^CD45RA^–^ (CD69^+^ from biopsies, CD69^–^ from blood) cell population (Tconv) were sorted into Eppendorfs containing 125 μL PBS. After sorting, 375 μL Trizol LS (Thermo Fisher Scientific) was added to each vial, and vials were stored at –80°C until RNA isolation.

### Suppression assays and cytokine measurement.

After sorting, peripheral blood, uTregs, and Tconv were washed in PBS and resuspended in basic medium with 10% human AB serum (Sanquin). Previously isolated and frozen healthy (HC) PBMC were labeled with 2 μM CellTrace Violet (Thermo Fisher Scientific) as described previously (128). Treg or Tconv populations were added to 15,000 HC PBMC at different ratios, and cells were coincubated for 4 days at 37°C. Supernatants were collected for cytokine measurement by multiplex assay before cells were stained with surface antibodies for CD3, CD4, and CD8 as described above and measured on a FACS Canto (BD Biosciences).

### Whole transcriptome sequencing.

For RNA isolation, the vials were thawed at room temperature, and 100 μL chloroform was added to each vial. The vials were shaken well and spun down at 12,000*g* for 15 minutes at 4°C. The aqueous phase was transferred into a new tube, and RNA was mixed with 1 μL of GlycoBlue (Invitrogen) and precipitated with 250 μL isopropanol. Samples were incubated at –20°C for 1 hour and subsequently spun down at 12,000*g* for 10 minutes. The supernatant was carefully discarded, and the RNA pellet was washed twice with 375 μL 75% ethanol. Vials were stored at –80°C until library preparation. Low-input RNA-seq libraries from biological sorted cell population replicates were prepared using the Cel-Seq2 Sample Preparation Protocol (129) and sequenced as 2 × 75 bp paired-end on a NextSeq 500 (Utrecht Sequencing Facility). The reads were demultiplexed and aligned to human cDNA reference using the BWA (0.7.13) (130). Multiple reads mapping to the same gene with the same unique molecular identifier (UMI, 6bp long) were counted as a single read.

### Statistics.

RNA-seq data were normalized per million reads per sample. Differentially expressed genes between the cell populations were identified using the DESeq2 package in R 3.5.1 (CRAN), with correction for donor batch (design = ~donor + cell population) and input of all genes. Genes with FDR *P*adj < 0.05 and |log_2_FC| > 0.5 were considered differentially expressed. PCA was performed in DESeq2 based on the constructed model, including donor correction. Pathway enrichment analysis was conducted in the Toppgene Suite publicly available online portal (https://toppgene.cchmc.org/enrichment.jsp), and pathways with Bonferroni-corrected *P* < 0.05 were considered statistically significant (131). For heatmap analysis, gene expression was mean centered and scaled per gene, and hierarchical clustering was performed with Ward’s method and Euclidian distance. GSEA (132) was conducted with Broad Institute software, by 1000 random permutations of the phenotypic subgroups to establish a null distribution of enrichment score, against which a normalized enrichment scores and multiple testing FDR-corrected *q* values were calculated. Gene sets with an FDR < 0.05 were considered significantly enriched. Gene sets were either obtained from provided data in publications or by analyzing raw data using GEO2R (NCBI tool) (133). An overview of used signatures is provided in Supplemental Table 3. For flow cytometric data, median fluorescence intensities (MFI) and percentages of positive cells were analyzed in FlowJo. For graphic representation, data were analyzed in GraphPad Prism. To assess significant differences on protein level between groups, 2-way ANOVA with Tukey’s post hoc test was used, and multiplicity-adjusted *P* < 0.05 were considered statistically significant.

The data sets generated for this study have been submitted to a public repository on GitHub ((https://github.com/JudithWienke/Human-uterine-Tregs; branch name, master; Commit ID, f788ba7f8163d7f892ce4069a2c9c304cf922902) The raw data files could not be submitted due to GDPR constraints, but any additional required data can be requested with the corresponding author.

### Study approval.

All patients received study information and signed informed consent before participation. This study was reviewed and approved by the local Institutional Ethical Review Board of the University Medical Center Utrecht (no. 16-198).

## Author contributions

LB recruited and included patients, and collected clinical data. JW, LB, RCS, and LMVDB performed all wet-lab experiments. MM performed the RNA-seq and helped with data analysis. JW performed all data analyses and wrote the manuscript. PGJN consulted on biopsy preparation, tissue integrity, and uterine T cell distribution and phenotype. BBVR and FVW supervised JW, LB, LMVDB, and RCS and were closely involved in setting up the study protocol, collecting data, conducting data analysis, and writing the manuscript. All authors critically revised the manuscript.

## Supplementary Material

Supplemental data

## Version 1 08/18/2020

In-Press Preview

## Version 2 09/17/2020

Electronic publication
