# Supplementary material for: Human Tregs at the materno-fetal interface show site-specific adaptation reminiscent of tumor Tregs
Source: JCI Insight. 2020 Sep 17;5(18):e137926. doi: 10.1172/jci.insight.137926 (PMC7526557; doi:10.1172/jci.insight.137926)
Supplement: Supplemental data [file jciinsight-5-137926-s091.pdf]

Supplementary Materials

- Fig. S1.uTregs derived from the maternal-fetal interface are bona fide maternal Tregs.
- Fig. S2. Enrichment of gene signatures of *in vitro* activated Tregs.
- Fig. S3. Tregs at the maternal-fetal interface share transcriptional adaptation with uTconv.
- Fig. S4. uTregs from the maternal-fetal interface share similarities were human Tregs from healthy tissue sites.
- Fig. S5. uTregs from the incision site are bona fide Tregs and have a tissue-resident profile.
- Table S1. Clinical characteristics of 20 human subjects undergoing caesarian section.
- Table S2. Antibodies used for sorting and flow cytometric analysis.
- Table S3. Gene signatures used for gene set enrichment analysis and overlap with uTreg signature.
- Table S4. Upregulated genes in the uTreg-specific core signature.
- Table S5. Downregulated genes in the uTreg-specific core signature.
- Table S6. Overlap of tumor-infiltrating Treg signatures with significantly upregulated genes in uTregs vs bTregs (% of overlapping genes/genes in signature).
- Table S7. Genes most often shared between 7 tumor-infiltrating Treg signatures.

Fig. S1. uTregs derived from the maternal-fetal interface are bona fide maternal Tregs.

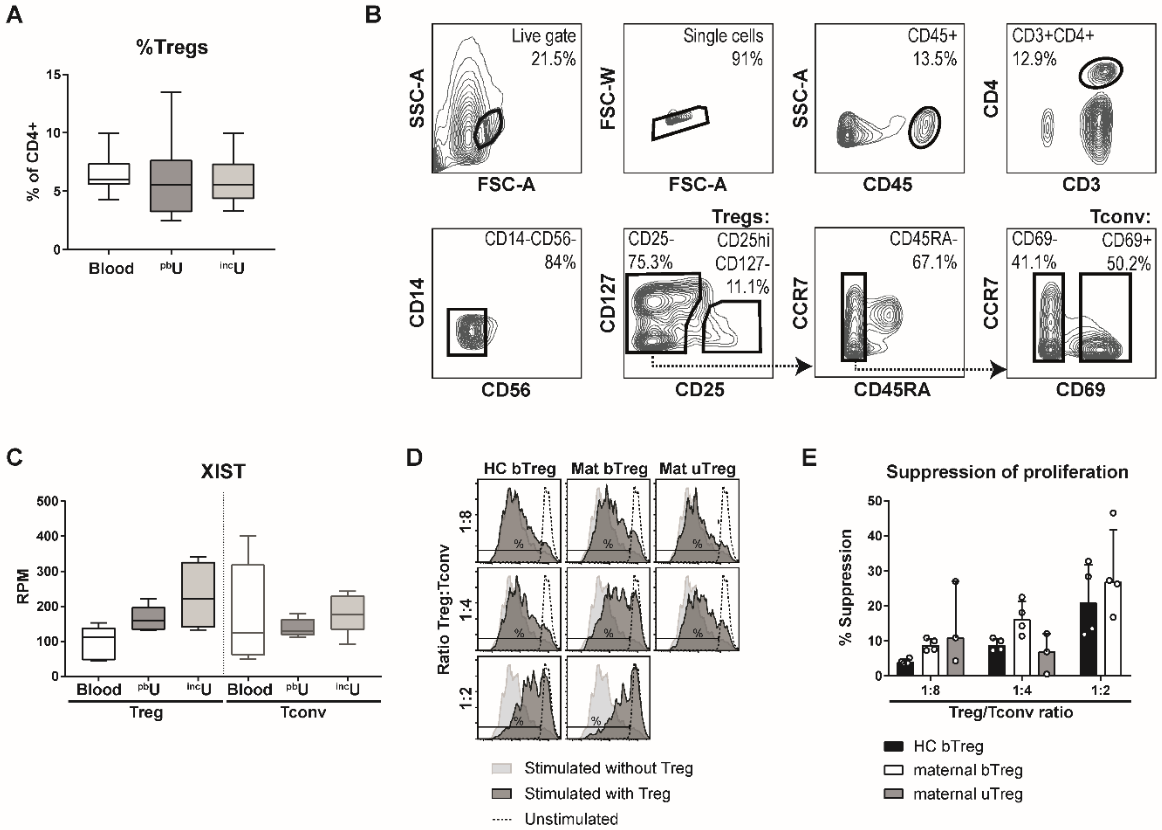

(A) Ex vivo frequency of CD25$^+$FOXP3$^+$ cells among CD4$^+$ T cells from blood, maternal-fetal interface (^{pb}U) and incision site (^{inc}U). **(B)** Sorting strategy for uTregs and uTconv in the uterus. **(C)** Expression of female-specific gene XIST in all sorted T cell subsets. **(D+E)** Suppresssion assay assessing proliferation of healthy CD4$^+$ T cells by CellTrace Violet dilution assay, after 4 days of coculture with healthy donor bTregs, maternal bTregs, or uTregs at a 1:8, 1:4 and 1:2 ratio (n=4).

Fig. S2. Enrichment of gene signatures of *in vitro* activated Tregs.

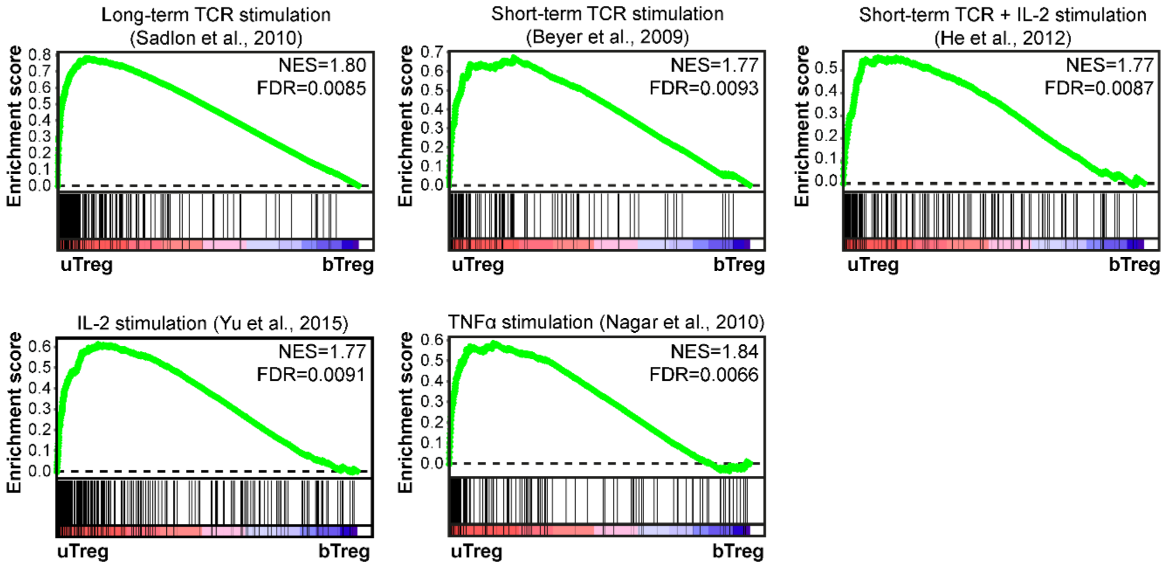

Gene set enrichment analysis with genes upregulated in Tregs stimulated *in vitro* with TCR stimulation, IL-2 or TNFα, comparing uTregs and bTregs. (81–85) NES = normalized enrichment score.

Fig. S3. Tregs at the maternal-fetal interface share transcriptional adaptation with uTconv.

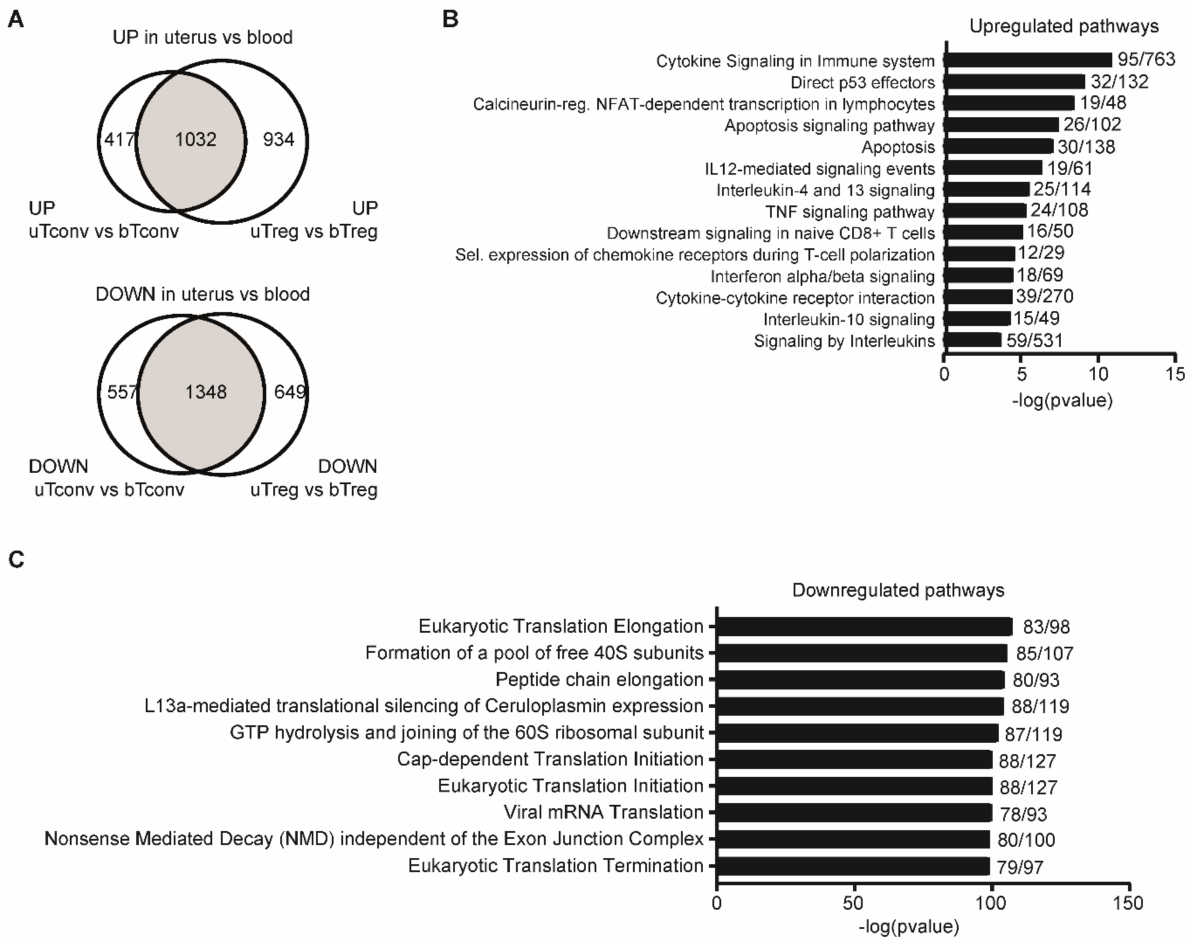

(A) Venn diagrams of upregulated and downregulated genes (padj<0.05 and |Log2FC|>0.05) shared between Tregs and $CD4^{+}$ Tconv from the maternal-fetal interface, compared to their blood-derived counterparts. **(B)** Pathway analysis (ToppGene pathways) of the 1032 shared upregulated genes in uTreg and uTconv. P-values<0.05 after Bonferroni correction were considered significant.
Pathway analysis (ToppGene Suite) of downregulated pathways shared among uTreg and uTconv. P-values<0.05 after Bonferroni correction were considered significant.

Fig. S4. uTregs from the maternal-fetal interface share similarities were human Tregs from healthy tissue sites.

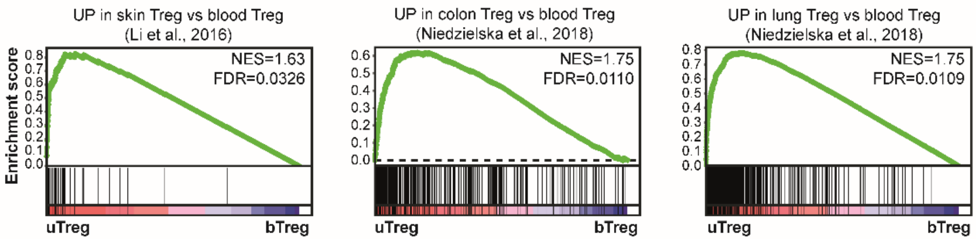

Gene set enrichment analysis with published genes which are significantly upregulated in tissue Tregs from healthy skin(15), colon(14) or lung(14) compared to blood Tregs, in uTregs versus bTregs.

Fig. S5. uTregs from the incision site are bona fide Tregs and have a tissue-resident profile.

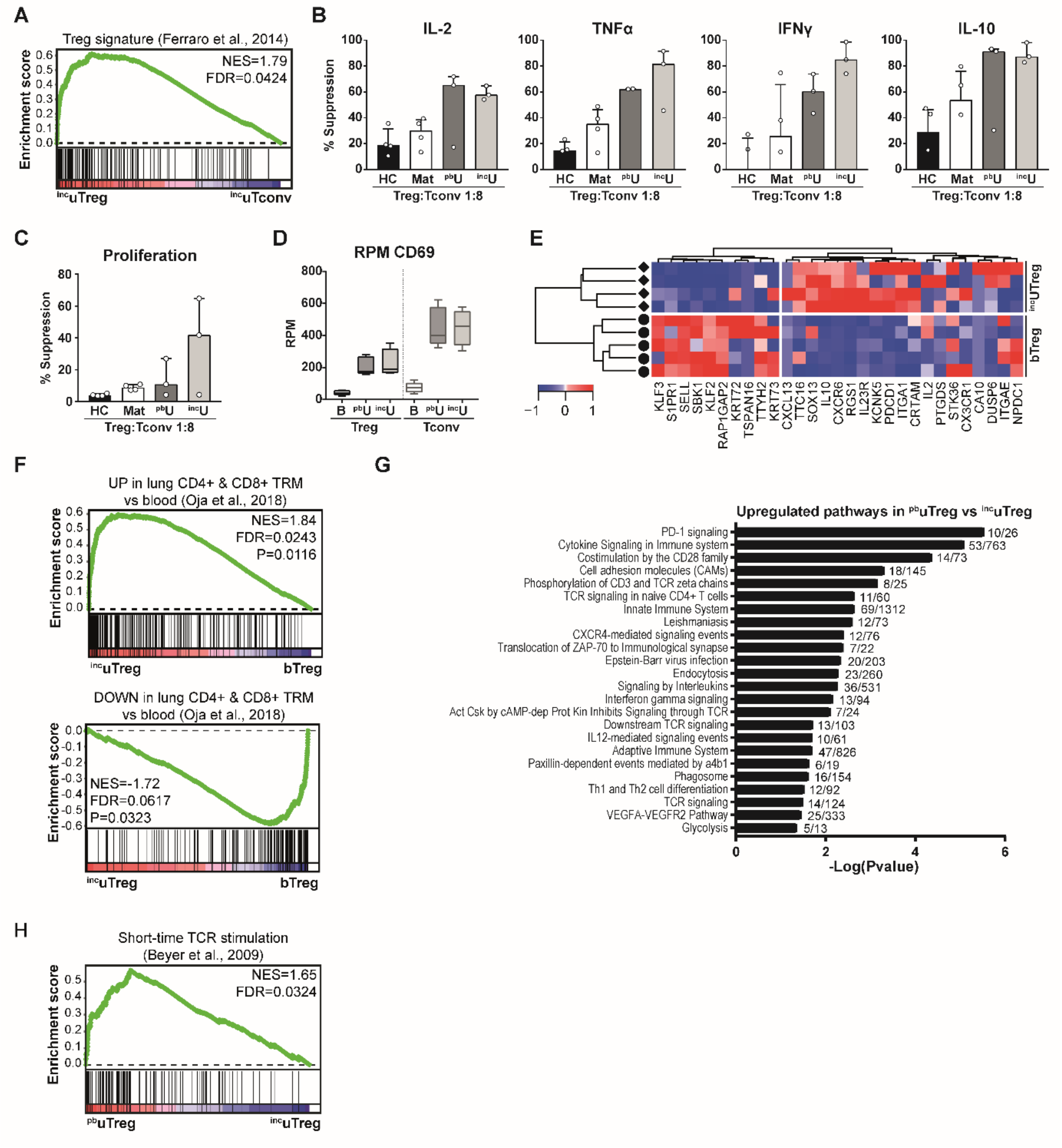

(A) GSEA with published Treg signature gene set comparing incuTreg and incuTconv.(71) **(B+C)** Suppresssion assay assessing proliferation of anti-CD3 stimulated (or unstimulated) healthy CD4$^+$ T cells by CellTrace Violet dilution assay **(C)** and cytokine production in the supernatant by multiplex immunoassay **(B)**, after 4 days of coculture with healthy donor bTregs, maternal bTregs, or uTregs at a 1:8, 1:4 and 1:2 ratio. **(D)** Gene expression of CD69. **(E)** Heatmap with published human core tissue-resident genes(4) in incuTregs vs bTregs **(F)**. Gene set enrichment analysis (GSEA) with published genes identifying human lung CD4$^+$ and CD8$^+$ TRM compared to blood memory cells; left panel(86)) and genes upregulated in skin CD4+ TRM compared to blood CD4+ T cells (right panel(15)), in incuTregs vs bTregs. NES = normalized enrichment score. **(G)** Pathway analysis (ToppGene Suite) with 558 genes upregulated in pbuTregs vs incuTregs. P-values<0.05 after Bonferroni correction were considered significant. **(H)** GSEA with gene set of upregulated genes in *in vitro* stimulated Tregs.(85) *NES = normalized enrichment score.*

Table S1. Clinical characteristics of 20 human subjects undergoing caesarian section.

**Maternal characteristics**	
Age (years), *mean (st.dev)*	34 (2.3)
White ethnicity, *n (%)*	19 (95%)
Gravida (n), *mean (st.dev)*	2.3 (1.0)
Para (n), *mean (st.dev)*	1.0 (0.7)
Nulliparous, *n (%)*	4 (20%)
Pregravid BMI (kg/m^2), *mean (st.dev)*	24.4 (5.2)

**Neonatal characteristics**	
Gestation age at birth (days), *mean (st.dev)*	275 (5)
Birthweight (grams), *mean (st.dev)*	3535 (480)
Birthweight (percentile), *mean (st.dev)*	60 (30)
FGR (birthweight <p10), *n (%)*	0 (0%)
LGA (birthweight >p95), *n (%)*	2 (10%)
Male sex, *n (%)*	6 (30%)
Apgar at 5 min post partum, *mean (st.dev)*	8.7 (0.8)
Apgar at 10 min post partum, *mean (st.dev)*	9.6 (0.9)

Table S2. Antibodies used for sorting and flow cytometric analysis

Cell sorting

Antibody	*Fluorochrome*	*Clone*	*Dilution (x)*	*Catalog no*	*Company*
CD69	FITC	FN50	25	130-113-523	Miltenyi
CCR7	PE	3D12	25	12-1979-42	eBioscience
CD4	PerCP-Cy5.5	RPA-T4	200	2102650	Sony Biotechnology
CD25	PE-Cy7	M-A251	25	557741	BD
CD127	AF647	HCD127	50	2356590	Sony Biotechnology
CD14	APC-Cy7	MphiP9	200	557831	BD
CD56?	PE-CF594	B159		562289	BD
CD45RA	PacBlue	HI100	200	2120590	Sony Biotechnology
CD3	BV510	OKT3	200	317332	Biolegend
CD45RA	BV711	HI30	400	304050	Biolegend

Flow cytometry

Surface antibody	*Fluorochrome*	*Clone*	*Dilution (x)*	*Catalog no*	*Company*
CCR5	FITC	2D7/CCR5	25	555992	BD
CCR8	PE	L263G8	100	360603	Biolegend
CD134 (OX-40)	PerCP-Cy5.5	Ber-ACT35	50	350010	Biolegend
CD137 (4-1BB)	APC	4B4-1	50	550890	BD
CD14	V500	M5E2	100	561391	BD
CD25	BV711	2A3	50	563159	BD
CD3	APC-eF780	UCHT1	400	47-0038-42	eBioscience
CD4	PE-Cy5	RPA-T4	50	555348	BD
CD56	BV510	HCD56	50	2191700	Sony Biotechnology
CD69	PE-Cy7	FN50	50	557745	BD
CXCR3	BV605	G025H7	12.5	2368640	Sony Biotechnology
CXCR5	PerCP-Cy5.5	TG2/CXCR5	200	TG2/CXCR5	AntibodyChain
GITR	FITC	#110416	25	FAB689F	R&D
HLA-DR	PerCP-Cy5.5	L243	100	307630	Biolegend
ICOS	APC	ISA3	25	17-9948-42	eBioscience
IL18R1	FITC	H44	12.5	11-7183-42	eBioscience
PD-1	APC	MIH4	12.5	558694	BD
TIGIT	PerCP-eF710	MBSA43	50	46-9200-42	eBioscience
Intracellular antibody	*Fluorochrome*	*Clone*	*Dilution (x)*	*Catalog no*	*Company*
CTLA-4	PE	BNI3	12.5	555853	BD
FOXP3	eFluor450	PCH101	50	48-4776-42	eBioscience
Ki67	AF647	B56	200	558615	BD
RORγt	APC	AFKJS-9	200	17-6988-82	eBioscience
T-bet	PE-CF594	O4-46	25	562467	BD
eBioscience™ Fixable Viability Dye	*eFluor506*		*300*	*65-0866-14*	*ThermoFisher*

Table S3. Gene signatures used for gene set enrichment analysis and overlap with uTreg signature

	**Reference**		**Geneset origin**		**Criteria for geneset**			
Figure	*Author, year*	*GEO NCBI*	*Organism*	*Tissue compartment*	*Publ/GEO2R*	*Pvalue/Padj*	*FC*	*Comparison*
1BC, S5A	Ferraro et al., 2014	-	Human	Peripheral blood	Publication			Treg signature vs Tconv
1I	Joller et al., 2014	-	Mouse	Spleen	Publication	P<0.05	FC>2	TIGIT+ vs TIGIT- Tregs
4B, S5E	Kumar et al., 2017	GSE94964	Human	Lung and spleen	Publication	Padj<0.05		CD69+ vs CD69- (shared among CD4+ and CD8+ from lung and spleen)
S4	Niedzielska et al., 2018	-	Human	Colon & lung vs peripheral blood	Publication	Padj<0.05	L2FC>2	Lung/Colon CD4+ vs blood CD4+
4C	Li et al., 2016	GSE74158	Human	Skin vs peripheral blood	GEO2R	Padj<0.05		Skin CD4+ vs blood CD4+
4C, S5F	Oja et al., 2018	-	Human	Lung vs peripheral blood	Publication	Padj<0.05		Shared between lung CD4+ and CD8+ TRM vs Blood TEM
5J	Tan et al., 2016	-	Mouse	Pancreas prediabetic T1D mice vs spleen	Publication	<0.05	FC>2	Splenic CXCR3+ versus CXCR3− Tregs (T-bet+)
6ABC	Dispirito et al., 2018	-	Mouse	VAT, muscle, Colon vs spleen	Publication	<0.05	FC>2	VAT/muscle/colon Tregs vs spleen Tregs
6DE,7AB	Plitas et al., 2016	-	Human	Breast cancer vs breast parenchyma vs peripheral blood	Publication	Padj<0.05		UP in breast cancer Treg vs breast cancer Tconv AND UP in breast cancer Treg vs Blood Treg
6DE,7AB	Toker et al., 2018	-	Human	Epithelial ovarian cancer (mostly high-grade serous) or melanoma	Publication	Padj<0.1		PD1intICOShi population (Tregs) vs Tconv
6DE,7AB, 9D	Tirosh et al., 2016	-	Human	Single cell melanoma	Publication	<0.01 (CD4) <0.05 (CD8)	FC>2	Meloma Treg vs CD4 Tconv and CD8 Tconv
6DE,7AB, 9D	Zheng et al., 2017	-	Human	Hepatocellular carcinoma single cell seq	Publ S3 Cluster	Padj<0.05	FC>2	Genes specific for Treg cluster identified by single cell seq
6DE,7AB, 9D	De Simone et al., 2016	-	Human	Colon and lung cancer vs colon and lung parenchyma vs blood vs Tconv	Publication			UP in TITR vs parenchyma vs blood vs Tconv
6DE,7AB, 9D	Pacella et al., 2018	-	Human	Liver cirrhosis and tumor (CT) or from the peripheral blood (PB) of patients with chronic HCV infection and hepatocellular carcinoma (HCC)	Publication		FC>2	Upregulated in Treg CT OX40+ versus Treg PB OX40-, and not in Treg CT OX40-, Treg PB OX40+ and all Tconv counteparts
6DE,7AB, 9D	Magnuson et al., 2018	-	Human	Colon cancer vs healthy colon	Publication			Tumor-Treg specific in mouse and human and correlation with FOXP3 expression
6DF	Li et al., 2016	GSE74158	Human	Skin vs peripheral blood	GEO2R	Padj<0.05		UP in skin Treg vs skin Tconv AND UP in skin Treg vs Blood Treg

Table S3. Gene signatures used for gene set enrichment analysis and overlap with uTreg signature

	**Reference**		**Geneset origin**		**Criteria for geneset**			
Figure	*Author, year*	*GEO NCBI*	*Organism*	*Tissue compartment*	*Publ/GEO2R*	*Pvalue/Padj*	*FC*	*Comparison*
6DF	Niedzielska et al., 2018	-	Human	Colon & lung vs peripheral blood	Publication	Padj<0.05	L2FC>2	UP in lung/colong Treg vs lung/colon Tconv AND UP in lung/colon Treg vs Blood Treg
7CD, 9E	Plitas et al., 2016	-	Human	Breast cancer vs breast parenchyma	Publication	Padj<0.05		UP in Breast cancer Tregs vs healthy breast parenchyma Tregs
7CD, 9E	Magnuson et al., 2018	-	Human	Colon cancer vs healthy colon	Publication			Mean overall fold change, or high fold change in at least two patients. Mean FoldChange > 2 and nominal p.value <0.02, or FoldChange >4 (UP), or <0.5 and <0.25 (DN).
S2	Nagar et al., 2010	GSE18893	Human	Peripheral blood	GEO2R	P<0.05	L2FC>0.5	50ng/mL TNFa stim vs unstim sorted Tregs (2 and 24 hrs combined in GEO2R)
S2	Yu et al., 2015	GSE49817	Human	Peripheral blood	GEO2R	Padj<0.05	UP FC>1.5, DOWN all FC	Sorted Treg stimulated with IL-2 24 hrsvs unstimulated Tregs
S2	He et al., 2012	GSE11292	Human	Peripheral blood	GEO2R	P<0.01	FC>10	Sorted Treg stim with anti-CD3/CD28 and IL-2 for 3 hours vs unstimulated Tregs
S2	Sadlon et al., 2010	GSE20934	Human	Cord blood	GEO2R	P<0.05	FC>2	Cord blood Treg expanded with dynabeads for 8 days + PMA/ionomycin vs resting Tregs at day 4
S2, S5H	Beyer et al., 2009	GSE16835	Human	Peripheral blood	GEO2R	Padj<0.05	FC>3	Tregs undergoing short time stimulation with anti-CD3/CD28 vs ex vivo Treg
S4	Li et al., 2016	GSE74158	Human	Skin vs peripheral blood	GEO2R	Padj<0.05		Skin Treg vs blood Treg
S4	Niedzielska et al., 2018	-	Human	Colon & lung vs peripheral blood	Publication	Padj<0.05	L2FC>2	Lung/Colon Treg vs blood Treg

Table S4. Upregulated genes in the uTreg-specific core signature.

uTreg core UP			UP vs bTreg			UP vs uTconv		
Gene	*Ensembl*	*baseMean*	*Log2(FC)*	*Pvalue*	*Padj*	*Log2(FC)*	*Pvalue*	*Padj*
**AC006064.4**	ENSG00000269968	153.4	1.22	1.09E-07	2.43E-06	0.75	9.81E-04	4.01E-02
**AC017002.1**	ENSG00000240350	24.1	2.69	6.15E-09	1.78E-07	3.09	3.19E-11	2.93E-08
**AC080038.1**	ENSG00000011028	5.4	3.80	1.31E-05	1.87E-04	3.12	6.80E-05	5.38E-03
**AC126603.1**	ENSG00000258628	76.9	1.49	1.00E-09	3.39E-08	1.09	4.15E-06	5.25E-04
**AC132825.2**	ENSG00000243655	163.7	0.89	5.90E-05	7.19E-04	0.73	8.51E-04	3.65E-02
**AC147651.4**	ENSG00000237181	7.6	1.49	3.93E-04	3.73E-03	1.78	2.30E-05	2.23E-03
**ACP5**	ENSG00000102575	28.6	1.58	2.39E-05	3.21E-04	1.92	2.74E-07	5.31E-05
**ADAMTS5**	ENSG00000154736	13.3	2.94	2.54E-07	5.30E-06	2.21	1.78E-05	1.85E-03
**ADPRH**	ENSG00000144843	8.3	1.56	7.12E-05	8.48E-04	1.92	1.15E-06	1.81E-04
**ADRM1**	ENSG00000130706	66.0	0.83	3.27E-04	3.20E-03	0.75	9.77E-04	4.00E-02
**AGTRAP**	ENSG00000177674	44.8	1.36	7.07E-09	2.01E-07	0.84	1.67E-04	1.10E-02
**AK4**	ENSG00000162433	5.6	4.35	6.96E-08	1.62E-06	1.91	4.74E-04	2.40E-02
**AKIRIN2**	ENSG00000135334	44.4	1.48	8.04E-07	1.51E-05	1.31	8.68E-06	1.02E-03
**AL390719.1**	ENSG00000217801	15.6	1.55	2.11E-07	4.49E-06	1.05	7.25E-05	5.67E-03
**ARHGAP11B**	ENSG00000187951	13.7	1.33	2.30E-05	3.09E-04	0.97	8.87E-04	3.75E-02
**ARID5B**	ENSG00000150347	203.1	0.88	3.06E-06	5.11E-05	1.04	3.32E-08	1.06E-05
**ARL3**	ENSG00000138175	26.3	0.93	6.30E-04	5.58E-03	0.88	7.74E-04	3.39E-02
**ASMTL**	ENSG00000169093	29.1	0.73	4.78E-03	3.08E-02	0.84	1.10E-03	4.28E-02
**ATP1B3**	ENSG00000069849	166.3	1.01	7.06E-08	1.64E-06	0.73	7.58E-05	5.89E-03
**ATP6V1B2**	ENSG00000147416	79.2	0.87	3.09E-06	5.15E-05	0.60	9.05E-04	3.80E-02
**B3GAT1**	ENSG00000109956	8.9	3.45	1.28E-08	3.49E-07	3.19	2.59E-08	8.67E-06
**B4GALT5**	ENSG00000158470	43.0	1.38	4.06E-10	1.47E-08	1.07	3.57E-07	6.56E-05
**BABAM2**	ENSG00000158019	40.5	1.07	4.84E-05	6.05E-04	1.10	2.14E-05	2.14E-03
**BATF**	ENSG00000156127	130.8	2.52	5.88E-16	6.25E-14	2.33	4.39E-14	8.43E-11
**BCL2L11**	ENSG00000153094	37.1	0.94	4.88E-05	6.10E-04	1.42	1.11E-09	6.88E-07
**BPGM**	ENSG00000172331	18.7	1.47	6.12E-06	9.56E-05	1.32	2.40E-05	2.31E-03
**BST2**	ENSG00000130303	59.7	1.66	7.32E-12	3.62E-10	0.82	3.35E-04	1.85E-02
**BTG3**	ENSG00000154640	97.0	2.33	1.13E-21	2.79E-19	0.91	7.65E-05	5.92E-03
**C15orf39**	ENSG00000167173	14.3	1.01	2.49E-03	1.79E-02	1.24	1.78E-04	1.14E-02
**CAMK1**	ENSG00000134072	22.1	2.67	2.90E-15	2.75E-13	1.75	6.98E-09	3.01E-06
**CAVIN3**	ENSG00000170955	17.0	3.95	5.62E-08	1.33E-06	2.07	4.95E-04	2.46E-02
**CCDC71L**	ENSG00000253276	15.8	0.98	6.83E-03	4.13E-02	1.38	1.46E-04	9.89E-03
**CCL3**	ENSG00000277632	4.7	3.15	5.15E-05	6.40E-04	2.37	6.85E-04	3.11E-02
**CCL3**	ENSG00000278567	5.1	3.74	9.33E-06	1.39E-04	3.49	1.01E-05	1.16E-03
**CCR1**	ENSG00000163823	12.1	8.33	3.68E-16	4.06E-14	2.05	2.57E-05	2.43E-03
**CD74**	ENSG00000019582	1077.4	1.00	2.59E-04	2.62E-03	1.55	1.62E-08	5.90E-06
**CD80**	ENSG00000121594	4.0	3.26	2.86E-06	4.81E-05	3.41	5.52E-07	9.56E-05
**CDKN1C**	ENSG00000273707	13.9	2.64	5.31E-06	8.39E-05	1.89	3.50E-04	1.91E-02
**CDKN2A**	ENSG00000147889	52.1	2.47	8.83E-14	6.42E-12	1.10	5.31E-04	2.59E-02
**CEBPB**	ENSG00000172216	63.7	1.73	9.23E-12	4.48E-10	0.89	2.17E-04	1.32E-02
**CFAP20**	ENSG00000070761	85.8	1.21	3.41E-07	6.90E-06	0.87	1.62E-04	1.07E-02
**CGA**	ENSG00000135346	4.2	6.00	3.41E-07	6.90E-06	4.78	3.00E-06	4.19E-04
**CHST11**	ENSG00000171310	117.7	0.86	2.00E-05	2.75E-04	0.87	1.22E-05	1.36E-03
**CHSY1**	ENSG00000131873	39.9	1.12	1.22E-07	2.71E-06	0.75	1.95E-04	1.22E-02
**COL9A2**	ENSG00000049089	10.0	3.83	3.30E-10	1.22E-08	2.68	7.45E-07	1.26E-04
**COMT**	ENSG00000093010	62.9	0.90	9.89E-08	2.23E-06	0.82	5.96E-07	1.02E-04
**COX17**	ENSG00000138495	95.7	0.90	2.08E-06	3.61E-05	0.76	4.03E-05	3.52E-03
**CRADD**	ENSG00000169372	6.6	2.29	3.04E-05	3.96E-04	1.95	1.28E-04	8.97E-03
**CSF1**	ENSG00000184371	48.9	3.53	4.64E-22	1.22E-19	1.33	5.67E-05	4.59E-03
**CTLA4**	ENSG00000163599	91.2	1.89	3.58E-09	1.09E-07	2.84	1.39E-18	5.85E-15
**CTNNA1**	ENSG00000044115	22.5	1.39	2.76E-07	5.70E-06	0.83	8.39E-04	3.62E-02
**CTSC**	ENSG00000109861	155.9	2.06	1.81E-16	2.11E-14	1.28	1.93E-07	4.08E-05
**CXCR6**	ENSG00000172215	65.7	3.97	9.30E-28	4.08E-25	1.34	4.86E-05	4.11E-03
**CXorf40A**	ENSG00000197620	33.9	0.96	8.49E-06	1.27E-04	0.98	2.66E-06	3.75E-04
**CXorf40B**	ENSG00000197021	22.0	0.98	3.29E-04	3.22E-03	1.26	3.42E-06	4.55E-04
**DNAJC12**	ENSG00000108176	7.3	2.82	3.85E-05	4.92E-04	2.13	8.35E-04	3.61E-02
**DPYSL2**	ENSG00000092964	37.6	1.04	5.08E-04	4.65E-03	1.42	2.10E-06	3.07E-04
**DUSP10**	ENSG00000143507	32.9	2.91	2.01E-25	7.34E-23	1.13	8.16E-07	1.37E-04
**DYNLL1**	ENSG00000088986	347.0	0.92	4.14E-06	6.68E-05	0.70	4.83E-04	2.43E-02

Table S4. Upregulated genes in the uTreg-specific core signature.

uTreg core UP			UP vs bTreg			UP vs uTconv		
Gene	*Ensembl*	*baseMean*	*Log2(FC)*	*Pvalue*	*Padj*	*Log2(FC)*	*Pvalue*	*Padj*
**EIF2AK3**	ENSG00000172071	11.5	1.65	3.04E-06	5.08E-05	1.01	1.02E-03	4.06E-02
**ELL2**	ENSG00000118985	12.2	2.11	5.86E-06	9.19E-05	1.40	1.17E-03	4.55E-02
**ENO1**	ENSG00000074800	971.5	1.54	1.27E-11	5.98E-10	0.74	1.08E-03	4.24E-02
**ENTPD1**	ENSG00000138185	72.2	0.50	2.68E-03	1.91E-02	0.88	1.39E-07	3.13E-05
**ERI1**	ENSG00000104626	26.5	1.43	6.70E-09	1.91E-07	1.03	9.45E-06	1.10E-03
**ETS2**	ENSG00000157557	17.9	2.36	1.88E-07	4.04E-06	2.21	4.31E-07	7.77E-05
**ETV7**	ENSG00000010030	27.6	1.40	4.05E-05	5.16E-04	1.56	3.97E-06	5.05E-04
**EVA1B**	ENSG00000142694	12.9	1.21	1.54E-03	1.19E-02	1.62	2.69E-05	2.53E-03
**FABP5**	ENSG00000164687	21.0	1.72	2.55E-06	4.35E-05	1.66	3.21E-06	4.34E-04
**FAM3C**	ENSG00000196937	13.2	2.39	7.45E-11	3.09E-09	1.56	1.21E-06	1.88E-04
**FGL2**	ENSG00000127951	13.2	1.50	7.55E-06	1.14E-04	1.24	8.51E-05	6.52E-03
**FKBP1A**	ENSG00000088832	236.1	0.94	1.02E-05	1.49E-04	0.81	1.26E-04	8.83E-03
**FKBP1C**	ENSG00000198225	18.5	0.95	3.00E-04	2.98E-03	1.29	9.63E-07	1.58E-04
**FOXB1**	ENSG00000171956	3.2	2.44	8.75E-04	7.40E-03	3.48	1.26E-05	1.40E-03
**FUCA2**	ENSG00000001036	19.3	1.52	2.65E-07	5.51E-06	0.97	3.56E-04	1.92E-02
**GADD45A**	ENSG00000116717	36.1	2.38	1.94E-09	6.19E-08	1.84	1.71E-06	2.60E-04
**GADD45G**	ENSG00000130222	17.4	6.43	1.48E-15	1.47E-13	1.88	3.63E-06	4.70E-04
**GAPDH**	ENSG00000111640	955.4	1.30	1.20E-09	4.00E-08	0.76	3.52E-04	1.91E-02
**GEM**	ENSG00000164949	10.8	8.39	1.33E-11	6.23E-10	5.29	3.64E-08	1.15E-05
**GLUD1**	ENSG00000148672	91.8	0.69	2.18E-04	2.26E-03	0.68	1.88E-04	1.19E-02
**GPAT3**	ENSG00000138678	6.8	2.33	3.17E-06	5.26E-05	1.48	4.98E-04	2.47E-02
**GPR137B**	ENSG00000077585	15.1	2.38	7.35E-13	4.49E-11	0.83	1.07E-03	4.21E-02
**GSTO1**	ENSG00000148834	85.1	0.80	2.46E-04	2.50E-03	0.74	6.33E-04	2.97E-02
**HAVCR2**	ENSG00000135077	25.3	1.40	1.94E-07	4.17E-06	0.85	6.14E-04	2.89E-02
**HES1**	ENSG00000114315	12.2	5.22	7.09E-08	1.65E-06	3.65	3.14E-07	5.87E-05
**HLA-A**	ENSG00000223980	729.5	0.70	1.28E-03	1.02E-02	0.75	5.52E-04	2.66E-02
**HLA-A**	ENSG00000224320	848.3	0.64	1.12E-03	9.07E-03	0.67	6.20E-04	2.91E-02
**HLA-A**	ENSG00000227715	787.4	0.66	1.87E-03	1.41E-02	0.71	8.26E-04	3.58E-02
**HLA-A**	ENSG00000235657	781.5	0.63	3.15E-03	2.18E-02	0.69	1.15E-03	4.48E-02
**HLA-DQB1**	ENSG00000225824	33.0	1.33	9.31E-04	7.78E-03	1.32	8.50E-04	3.65E-02
**HLA-DRA**	ENSG00000204287	12.0	1.31	7.35E-03	4.37E-02	2.75	1.18E-07	2.78E-05
**HLA-DRA**	ENSG00000227993	11.6	1.41	5.81E-03	3.61E-02	3.35	2.57E-09	1.39E-06
**HLA-DRA**	ENSG00000228987	13.6	1.56	1.52E-03	1.18E-02	3.34	4.79E-10	3.27E-07
**HLA-DRA**	ENSG00000234794	13.2	1.88	3.23E-05	4.18E-04	3.94	6.25E-14	1.10E-10
**HLA-DRB1**	ENSG00000228080	111.4	1.54	3.13E-03	2.17E-02	2.75	2.70E-07	5.27E-05
**HLA-DRB1**	ENSG00000229074	113.7	1.84	3.53E-04	3.42E-03	2.88	7.09E-08	1.92E-05
**HLA-DRB4**	ENSG00000227357	20.9	1.55	1.97E-03	1.48E-02	2.20	1.43E-05	1.55E-03
**HLA-DRB4**	ENSG00000231021	21.3	1.88	7.43E-05	8.79E-04	2.57	8.73E-08	2.25E-05
**HNRNPLL**	ENSG00000143889	87.6	0.79	1.13E-04	1.26E-03	0.71	4.60E-04	2.35E-02
**IFT27**	ENSG00000100360	21.3	0.70	5.11E-03	3.25E-02	0.90	2.45E-04	1.45E-02
**IGFLR1**	ENSG00000126246	59.4	0.81	1.88E-05	2.61E-04	1.10	4.67E-09	2.19E-06
**IKZF4**	ENSG00000123411	26.2	0.81	6.43E-03	3.93E-02	1.53	5.39E-07	9.42E-05
**IL10**	ENSG00000136634	28.3	3.05	8.34E-10	2.84E-08	2.56	1.05E-07	2.55E-05
**IL1R1**	ENSG00000115594	22.0	1.47	9.82E-06	1.45E-04	1.19	1.91E-04	1.20E-02
**IL1R2**	ENSG00000115590	12.2	4.22	7.54E-04	6.52E-03	4.25	6.07E-04	2.88E-02
**IL1RAP**	ENSG00000196083	18.1	0.87	4.66E-03	3.01E-02	0.97	1.31E-03	4.91E-02
**IL1RN**	ENSG00000136689	5.1	3.51	6.95E-05	8.31E-04	4.56	1.72E-06	2.60E-04
**IL2RA**	ENSG00000134460	114.7	0.98	4.67E-05	5.87E-04	3.11	2.43E-35	5.14E-31
**IL2RB**	ENSG00000100385	224.5	1.96	7.34E-12	3.62E-10	1.22	1.76E-05	1.84E-03
**ITGAM**	ENSG00000169896	19.0	3.14	5.08E-08	1.22E-06	3.07	5.50E-08	1.59E-05
**JMJD4**	ENSG00000081692	43.8	1.02	1.03E-06	1.88E-05	0.66	8.98E-04	3.79E-02
**JOSD2**	ENSG00000161677	29.9	1.14	9.96E-06	1.47E-04	0.85	5.78E-04	2.76E-02
**KAT2B**	ENSG00000114166	31.9	1.39	4.80E-08	1.16E-06	0.81	6.97E-04	3.12E-02
**KCNK5**	ENSG00000164626	5.1	4.87	3.11E-09	9.64E-08	2.59	4.64E-07	8.23E-05
**KDM2A**	ENSG00000173120	123.4	1.05	7.55E-07	1.42E-05	0.69	9.00E-04	3.79E-02
**KLHL2**	ENSG00000109466	12.8	1.18	4.52E-04	4.22E-03	1.12	5.40E-04	2.61E-02
**LAG3**	ENSG00000089692	25.1	4.17	4.64E-17	6.07E-15	1.59	1.61E-04	1.07E-02
**LAPTM4B**	ENSG00000104341	47.7	1.76	1.25E-12	7.21E-11	0.80	5.00E-04	2.47E-02
**LAYN**	ENSG00000204381	10.0	2.84	1.24E-08	3.38E-07	3.48	1.82E-11	1.83E-08

Table S4. Upregulated genes in the uTreg-specific core signature.

uTreg core UP			UP vs bTreg			UP vs uTconv		
Gene	*Ensembl*	*baseMean*	*Log2(FC)*	*Pvalue*	*Padj*	*Log2(FC)*	*Pvalue*	*Padj*
**LGALS1**	ENSG00000100097	340.4	2.90	6.11E-17	7.82E-15	1.39	5.08E-05	4.21E-03
**LGALS3**	ENSG00000131981	197.5	2.00	7.97E-14	5.84E-12	1.26	1.72E-06	2.60E-04
**LINC01943**	ENSG00000280721	29.5	2.91	2.85E-10	1.08E-08	2.56	1.46E-08	5.56E-06
**LINC02195**	ENSG00000236481	6.4	2.65	4.25E-07	8.39E-06	4.54	3.45E-12	4.29E-09
**LRRC32**	ENSG00000137507	8.4	2.49	3.39E-04	3.31E-03	4.27	3.07E-08	1.01E-05
**LTA**	ENSG00000231408	9.2	2.43	9.72E-08	2.20E-06	1.33	3.39E-04	1.87E-02
**MAF**	ENSG00000178573	290.5	0.98	1.98E-06	3.45E-05	1.07	2.51E-07	4.95E-05
**MAPKAPK3**	ENSG00000114738	62.8	1.97	5.20E-16	5.60E-14	0.72	1.28E-03	4.86E-02
**MAST4**	ENSG00000069020	181.9	1.25	2.74E-11	1.23E-09	0.96	1.79E-07	3.88E-05
**MB21D2**	ENSG00000180611	19.9	3.33	6.67E-11	2.78E-09	1.68	1.70E-04	1.11E-02
**MCM6**	ENSG00000076003	29.5	0.77	7.50E-04	6.49E-03	0.95	2.43E-05	2.32E-03
**MGST2**	ENSG00000085871	15.3	0.87	1.58E-03	1.22E-02	1.65	1.19E-08	4.67E-06
**MIF-AS1**	ENSG00000218537	60.7	0.79	2.99E-04	2.97E-03	0.73	6.10E-04	2.88E-02
**MTREX**	ENSG00000039123	91.6	2.46	1.62E-14	1.38E-12	1.68	7.36E-08	1.94E-05
**MT-RNR1**	ENSG00000211459	4450.1	1.73	1.53E-20	3.19E-18	0.60	1.28E-03	4.86E-02
**MTRNR2L1**	ENSG00000256618	2315.5	1.43	4.17E-16	4.53E-14	0.60	6.77E-04	3.09E-02
**MTRNR2L10**	ENSG00000256045	141.2	1.65	1.64E-13	1.14E-11	0.69	1.24E-03	4.76E-02
**MYO1E**	ENSG00000157483	8.8	3.20	1.51E-11	6.97E-10	2.40	1.67E-09	9.51E-07
**MYO7A**	ENSG00000137474	10.7	1.67	1.49E-04	1.62E-03	1.90	1.54E-05	1.64E-03
**NAB1**	ENSG00000138386	13.0	2.29	1.79E-09	5.76E-08	1.86	6.71E-08	1.84E-05
**NAMPT**	ENSG00000105835	64.2	3.21	2.78E-59	5.23E-56	1.81	3.15E-29	2.22E-25
**NAMPTP1**	ENSG00000229644	17.8	4.02	1.48E-23	4.55E-21	2.75	5.12E-18	1.80E-14
**NCF4**	ENSG00000100365	43.6	0.78	1.81E-03	1.38E-02	1.15	4.20E-06	5.29E-04
**NDFIP2**	ENSG00000102471	26.4	3.80	5.28E-20	1.00E-17	1.32	9.62E-05	7.21E-03
**NDUFV2**	ENSG00000178127	124.3	0.60	1.60E-03	1.24E-02	0.60	1.26E-03	4.82E-02
**NFIL3**	ENSG00000165030	18.9	5.24	6.56E-17	8.27E-15	2.36	1.15E-07	2.76E-05
**NINJ1**	ENSG00000131669	109.4	2.23	7.31E-17	9.16E-15	1.18	5.11E-06	6.31E-04
**NMB**	ENSG00000197696	13.6	2.25	2.23E-09	7.02E-08	1.61	2.46E-06	3.51E-04
**NR4A3**	ENSG00000119508	39.3	7.22	7.00E-18	9.99E-16	0.80	5.07E-04	2.50E-02
**OGG1**	ENSG00000114026	39.8	1.04	4.55E-07	8.94E-06	0.66	6.41E-04	2.98E-02
**OTUD1**	ENSG00000165312	33.1	1.41	5.30E-05	6.56E-04	1.63	2.61E-06	3.70E-04
**PARPBP**	ENSG00000185480	13.3	1.55	2.16E-03	1.59E-02	1.70	6.88E-04	3.11E-02
**PDCD1**	ENSG00000276977	48.4	4.09	8.79E-26	3.41E-23	1.16	3.57E-04	1.92E-02
**PDGFA**	ENSG00000197461	11.0	1.65	1.48E-05	2.09E-04	1.39	9.53E-05	7.17E-03
**PELI1**	ENSG00000197329	38.8	1.31	3.30E-06	5.42E-05	1.96	7.41E-12	8.24E-09
**PGAM1P8**	ENSG00000255200	37.6	1.24	1.04E-07	2.34E-06	0.70	1.33E-03	4.94E-02
**PGK1**	ENSG00000102144	412.2	1.33	3.42E-10	1.26E-08	0.81	1.09E-04	7.95E-03
**PGM2L1**	ENSG00000165434	96.0	1.13	1.90E-08	5.04E-07	0.82	2.90E-05	2.69E-03
**PHLDA1**	ENSG00000139289	302.4	4.72	2.67E-72	1.82E-68	0.77	1.22E-03	4.68E-02
**PHLDA2**	ENSG00000181649	10.3	4.91	2.64E-12	1.41E-10	1.68	6.70E-05	5.34E-03
**PHLDA2**	ENSG00000274538	10.6	4.59	1.19E-13	8.49E-12	1.90	2.36E-06	3.39E-04
**PHTF2**	ENSG00000006576	43.3	1.65	1.03E-15	1.05E-13	0.67	2.62E-04	1.52E-02
**PIGT**	ENSG00000124155	43.2	0.70	1.85E-03	1.40E-02	0.83	1.71E-04	1.11E-02
**PIM3**	ENSG00000198355	173.1	2.55	3.74E-17	4.95E-15	1.04	4.16E-04	2.19E-02
**PKM**	ENSG00000067225	645.7	1.18	6.06E-06	9.47E-05	1.22	3.07E-06	4.27E-04
**PLPP1**	ENSG00000067113	55.9	3.51	7.01E-12	3.50E-10	2.17	1.10E-05	1.24E-03
**PMAIP1**	ENSG00000141682	41.3	2.33	4.27E-14	3.29E-12	1.17	4.97E-05	4.14E-03
**PMVK**	ENSG00000163344	40.2	1.04	2.94E-06	4.94E-05	0.69	1.26E-03	4.80E-02
**PRDM1**	ENSG00000057657	221.3	0.75	7.56E-05	8.92E-04	1.12	3.43E-09	1.72E-06
**PRDX1**	ENSG00000117450	77.6	0.57	3.67E-03	2.47E-02	0.72	2.27E-04	1.35E-02
**PRNP**	ENSG00000171867	62.5	1.90	2.57E-10	9.77E-09	0.92	1.35E-03	4.99E-02
**PSMD1**	ENSG00000173692	78.1	0.56	6.80E-04	5.95E-03	0.53	9.62E-04	3.97E-02
**PSMD8**	ENSG00000099341	126.4	0.51	3.59E-04	3.47E-03	0.59	3.06E-05	2.83E-03
**PTP4A3**	ENSG00000184489	9.6	1.72	6.52E-04	5.75E-03	1.80	2.79E-04	1.59E-02
**PTP4A3**	ENSG00000275575	7.4	1.66	3.52E-03	2.40E-02	1.98	5.20E-04	2.55E-02
**PTTG1**	ENSG00000164611	34.7	1.40	1.14E-05	1.64E-04	1.55	1.08E-06	1.71E-04
**PXK**	ENSG00000168297	14.5	1.14	1.04E-04	1.18E-03	0.95	6.53E-04	3.02E-02
**RAB10**	ENSG00000084733	52.4	0.92	4.96E-07	9.67E-06	0.60	5.66E-04	2.70E-02
**RAC1**	ENSG00000136238	238.4	0.62	3.09E-04	3.06E-03	0.66	9.74E-05	7.27E-03

Table S4. Upregulated genes in the uTreg-specific core signature.

uTreg core UP			UP vs bTreg			UP vs uTconv		
Gene	*Ensembl*	*baseMean*	*Log2(FC)*	*Pvalue*	*Padj*	*Log2(FC)*	*Pvalue*	*Padj*
**RBKS**	ENSG00000171174	7.1	2.49	5.12E-06	8.13E-05	1.79	2.02E-04	1.24E-02
**RCAN2**	ENSG00000172348	10.1	7.23	3.94E-12	2.04E-10	2.78	3.20E-05	2.93E-03
**RDH10**	ENSG00000121039	21.8	2.01	4.25E-08	1.04E-06	2.20	1.49E-09	8.77E-07
**RHBDD2**	ENSG00000005486	119.4	1.02	3.31E-05	4.29E-04	0.78	1.35E-03	4.99E-02
**RHPN2**	ENSG00000131941	87.0	1.20	9.09E-11	3.73E-09	1.07	4.40E-09	2.15E-06
**RNF187**	ENSG00000168159	79.1	0.81	2.16E-04	2.24E-03	0.75	5.21E-04	2.55E-02
**SAT1**	ENSG00000130066	194.6	0.79	1.03E-03	8.44E-03	0.94	1.07E-04	7.82E-03
**SDC4**	ENSG00000124145	39.0	3.53	1.11E-22	3.14E-20	1.83	1.47E-08	5.56E-06
**SDF4**	ENSG00000078808	115.6	0.85	9.96E-05	1.14E-03	0.77	3.34E-04	1.85E-02
**SEC14L1**	ENSG00000129657	64.4	0.88	1.17E-04	1.30E-03	0.76	7.62E-04	3.35E-02
**SETBP1**	ENSG00000152217	4.7	3.11	6.49E-06	1.01E-04	1.83	7.08E-04	3.16E-02
**SGMS1**	ENSG00000198964	21.7	0.86	1.02E-03	8.41E-03	1.19	5.65E-06	6.90E-04
**SIGLEC17P**	ENSG00000171101	15.4	5.68	5.02E-15	4.70E-13	3.41	3.33E-10	2.35E-07
**SIPA1L1**	ENSG00000197555	36.4	1.50	4.21E-09	1.25E-07	1.07	8.86E-06	1.03E-03
**SLC16A1**	ENSG00000155380	9.0	1.59	3.33E-05	4.31E-04	1.49	4.48E-05	3.86E-03
**SLC27A2**	ENSG00000140284	4.6	4.87	5.27E-07	1.02E-05	3.45	8.25E-06	9.79E-04
**SLC5A3**	ENSG00000198743	33.6	1.96	2.69E-16	3.06E-14	0.69	6.94E-04	3.12E-02
**SLC7A5**	ENSG00000103257	72.9	3.63	2.77E-23	8.34E-21	1.45	1.83E-05	1.88E-03
**SLCO4A1**	ENSG00000101187	4.8	3.53	5.97E-07	1.15E-05	2.08	2.27E-04	1.35E-02
**SMOX**	ENSG00000088826	6.0	4.83	5.65E-08	1.34E-06	2.20	1.63E-04	1.07E-02
**SNAP47**	ENSG00000143740	41.5	2.09	3.46E-12	1.81E-10	1.16	4.04E-05	3.52E-03
**SNX5**	ENSG00000089006	76.3	0.52	2.92E-04	2.91E-03	0.56	5.66E-05	4.59E-03
**SNX9**	ENSG00000130340	38.7	1.64	7.13E-07	1.35E-05	1.38	2.01E-05	2.04E-03
**SOX4**	ENSG00000124766	12.6	1.89	2.02E-04	2.12E-03	2.21	1.46E-05	1.58E-03
**SPATS2L**	ENSG00000196141	90.6	1.55	1.12E-19	2.01E-17	0.81	4.40E-07	7.87E-05
**SRGN**	ENSG00000122862	1731.7	2.71	1.15E-46	1.36E-43	0.69	2.21E-04	1.33E-02
**SURF4**	ENSG00000148248	73.2	1.49	3.46E-10	1.27E-08	0.74	1.20E-03	4.64E-02
**SUSD6**	ENSG00000100647	70.5	1.14	3.47E-06	5.68E-05	0.80	9.72E-04	3.99E-02
**SYT11**	ENSG00000132718	44.7	0.69	4.55E-04	4.24E-03	1.08	4.90E-08	1.48E-05
**TFRC**	ENSG00000072274	78.9	1.02	3.36E-08	8.38E-07	1.28	3.30E-12	4.29E-09
**TGIF2-RAB5IF**	ENSG00000259399	8.7	0.98	7.50E-03	4.44E-02	1.24	6.52E-04	3.02E-02
**TMED3**	ENSG00000166557	67.3	0.59	2.95E-04	2.94E-03	0.77	1.49E-06	2.30E-04
**TMEM173**	ENSG00000184584	225.7	0.87	6.27E-06	9.77E-05	0.65	6.75E-04	3.09E-02
**TNFRSF13B**	ENSG00000240505	4.5	3.63	6.63E-06	1.02E-04	6.76	1.88E-09	1.05E-06
**TNFRSF18**	ENSG00000186891	58.6	4.15	3.77E-22	1.03E-19	2.19	3.20E-08	1.04E-05
**TNFRSF1B**	ENSG00000028137	539.9	1.11	9.86E-06	1.46E-04	1.20	1.91E-06	2.85E-04
**TNFRSF4**	ENSG00000186827	95.2	3.07	7.53E-17	9.38E-15	2.82	1.04E-14	2.45E-11
**TNFRSF8**	ENSG00000120949	2.8	2.44	3.66E-03	2.47E-02	4.56	2.11E-05	2.12E-03
**TNFRSF9**	ENSG00000049249	74.3	1.55	9.01E-11	3.70E-09	1.13	1.06E-06	1.69E-04
**TNIP2**	ENSG00000168884	77.4	0.95	4.76E-04	4.41E-03	0.92	6.80E-04	3.09E-02
**TNS3**	ENSG00000136205	19.4	2.25	2.74E-12	1.45E-10	0.92	4.31E-04	2.26E-02
**TOX2**	ENSG00000124191	14.5	2.31	1.08E-04	1.22E-03	2.66	8.41E-06	9.93E-04
**TP53INP2**	ENSG00000078804	15.0	4.26	1.64E-14	1.39E-12	1.03	9.85E-04	4.01E-02
**TPI1**	ENSG00000111669	218.1	1.50	2.84E-08	7.24E-07	0.99	2.46E-04	1.45E-02
**TPP1**	ENSG00000166340	187.5	0.66	1.12E-03	9.09E-03	0.68	6.95E-04	3.12E-02
**TRAF1**	ENSG00000056558	64.5	1.45	4.55E-08	1.11E-06	1.13	1.29E-05	1.42E-03
**TRAF3**	ENSG00000131323	27.8	0.58	7.40E-03	4.39E-02	0.84	1.07E-04	7.82E-03
**TRPS1**	ENSG00000104447	17.6	1.09	3.84E-04	3.66E-03	0.94	1.34E-03	4.98E-02
**TSPAN13**	ENSG00000106537	5.8	6.96	5.27E-06	8.34E-05	5.08	1.82E-04	1.16E-02
**TSPAN17**	ENSG00000048140	25.6	0.84	2.46E-03	1.77E-02	0.95	5.64E-04	2.70E-02
**TTYH3**	ENSG00000136295	5.3	2.36	6.21E-05	7.51E-04	1.80	9.18E-04	3.84E-02
**U62317.1**	ENSG00000272666	5.2	3.30	5.54E-07	1.07E-05	1.82	9.99E-04	4.04E-02
**UBASH3B**	ENSG00000154127	30.9	1.24	1.72E-06	3.03E-05	1.17	3.10E-06	4.28E-04
**VDR**	ENSG00000111424	67.0	1.87	2.58E-13	1.73E-11	1.92	3.81E-14	8.05E-11
**VMP1**	ENSG00000062716	123.1	0.53	9.93E-05	1.13E-03	0.56	3.13E-05	2.88E-03
**ZBED2**	ENSG00000177494	5.9	5.64	8.61E-11	3.54E-09	3.31	2.96E-10	2.16E-07
**ZBTB32**	ENSG00000011590	5.6	2.22	7.03E-04	6.13E-03	3.74	3.04E-07	5.74E-05
**ZNF282**	ENSG00000170265	34.4	2.44	4.55E-10	1.63E-08	2.04	8.74E-08	2.25E-05
**ZNRF1**	ENSG00000186187	29.0	2.72	6.38E-15	5.90E-13	1.37	1.31E-05	1.43E-03

Table S5. Downregulated genes in the uTreg-specific core signature.

uTreg core DOWN			DOWN vs bTreg			DOWN vs uTconv		
Gene	*Ensembl*	*baseMean*	*Log2(FC)*	*Pvalue*	*Padj*	*Log2(FC)*	*Pvalue*	*Padj*
**ABLIM1**	ENSG00000099204	131.6	-2.07	5.82E-32	3.36E-29	-0.84	3.61E-06	4.70E-04
**AL157935.1**	ENSG00000227218	19.5	-1.55	4.81E-08	1.16E-06	-1.00	5.53E-04	2.66E-02
**APBA2**	ENSG00000034053	17.8	-1.10	1.54E-03	1.20E-02	-1.44	1.55E-05	1.64E-03
**ATF7IP2**	ENSG00000166669	56.4	-1.44	1.84E-12	1.02E-10	-0.95	3.88E-06	4.97E-04
**BEX2**	ENSG00000133134	42.6	-2.54	4.66E-20	8.90E-18	-1.20	3.25E-05	2.96E-03
**CCR7**	ENSG00000126353	605.6	-1.83	2.50E-13	1.68E-11	-0.94	1.80E-04	1.15E-02
**GCSAM**	ENSG00000174500	29.7	-1.25	4.56E-04	4.25E-03	-1.96	8.19E-09	3.39E-06
**GIMAP4**	ENSG00000133574	251.1	-1.63	3.65E-23	1.07E-20	-0.81	1.02E-06	1.65E-04
**GIMAP7**	ENSG00000179144	352.4	-2.13	1.31E-21	3.20E-19	-0.74	1.01E-03	4.06E-02
**IL7R**	ENSG00000168685	1201.0	-0.71	8.53E-03	4.92E-02	-1.62	1.27E-09	7.65E-07
**ITGA6**	ENSG00000091409	53.7	-2.28	4.53E-20	8.72E-18	-0.90	5.64E-04	2.70E-02
**LDLRAP1**	ENSG00000157978	166.2	-2.14	8.14E-30	4.19E-27	-0.62	1.32E-03	4.91E-02
**LEF1**	ENSG00000138795	241.9	-3.31	2.49E-57	4.22E-54	-0.77	3.16E-04	1.78E-02
**LINC02273**	ENSG00000245954	42.9	-1.82	2.72E-08	6.96E-07	-1.81	2.20E-08	7.49E-06
**MGAT4A**	ENSG00000071073	100.1	-1.09	5.05E-09	1.48E-07	-1.07	5.30E-09	2.38E-06
**PLAC8**	ENSG00000145287	136.7	-1.02	1.12E-06	2.04E-05	-1.09	1.17E-07	2.78E-05
**PRKCB**	ENSG00000166501	114.2	-1.23	1.27E-12	7.30E-11	-0.85	1.19E-06	1.86E-04
**RARRES3**	ENSG00000133321	208.5	-0.62	4.25E-04	4.00E-03	-0.70	5.82E-05	4.67E-03
**RBL2**	ENSG00000103479	105.8	-0.96	7.38E-08	1.71E-06	-0.64	3.14E-04	1.78E-02
**SATB1**	ENSG00000182568	163.1	-0.80	1.10E-03	8.91E-03	-1.26	2.07E-07	4.34E-05
**TCF7**	ENSG00000081059	493.4	-1.75	1.84E-25	6.80E-23	-1.11	4.86E-11	4.28E-08
**TTC39C**	ENSG00000168234	158.1	-1.36	6.25E-25	2.18E-22	-0.71	1.30E-07	2.98E-05
**TTC9**	ENSG00000133985	40.7	-0.99	8.71E-04	7.37E-03	-1.15	9.23E-05	7.02E-03

Table S6. Overlap of tumor-infiltrating Treg signatures with significantly upregulated genes in uTregs vs bTregs (% of overlapping genes/genes in signature).

Overlap between uTregs and tumor-infiltrating Treg signatures						
Tirosh et al	Zheng et al	De Simone et al.	Pacella et al.	Toker et al.	Magnuson et al.	Plitas et al.
75/172 (44%)	*225/401 (56%)*	*92/309 (30%)*	*91/211 (43%)*	*42/124 (34%)*	*70/108 (65%)*	*105/423 (25%)*
ACP5	ACP5	ACAA2	ACP5	ARHGEF12	ANXA4	ACSL4
AGTRAP	ACSL4	ACP5	ACTG2	BCL2L1	ARHGEF12	ACTG2
ANXA2	ACTN4	ACSL4	AKAP13	CD80	ATF3	ADPRH
B4GALT1	AKIRIN2	ACTG2	AKIRIN2	COL5A1	BATF	AKAP5
BATF	APOBEC3C	ADPRH	APOBEC3C	CRADD	BCL2L1	ASB2
BIRC3	APOBEC3G	AKAP5	ARID5B	CREB3L2	CAPG	ATP6V1A
BST2	ARHGEF12	ARHGEF12	ATP6V1B2	CTSC	CCR5	ATP6V1C2
C17orf49	ARID5B	ARNTL2	ATP6V1D	DPYSL2	CD74	AURKA
C3AR1	ARPP19	AURKA	B3GAT1	GADD45A	CD80	BCL2
CAP1	ASB2	BATF	BCL2L1	GCNT1	CDKN1A	C3AR1
CCND2	ASXL2	BCL2L1	CAMK1	HIP1	CPD	CCND2
CD79B	ATP1B1	CD7	CCDC6	ICOS	CREB3L2	CCRL2
CLIC1	ATP6V1C2	CDK6	CCND2	IL1R1	CREM	CD40LG
CMC2	BATF	CEACAM1	CD2	IL1R2	CTNNA1	CD7
COX17	BCL2L1	CGA	CD4	IL2RA	CTSH	CD79B
CRADD	BIRC3	COL9A2	CD63	KAT2B	CXCR3	CD80
CTLA4	BST2	CRADD	CD7	KBTBD8	CYFIP1	CDK6
CTSC	BTG3	CREB3L2	CD74	KSR1	DUSP4	CGA
CXCR3	C21orf91	CSF1	CD79B	LAPTM4B	ENTPD1	CKS2
CXCR6	C3AR1	CTLA4	CDK2AP1	LAYN	FAM126A	COL9A2
DPYSL2	CCL20	CTSC	CEACAM1	LRRC32	FAM129A	CORO1C
EIF4A1	CCND2	DPYSL2	CLIC1	LTA	FNDC3B	COX17
ENO1	CCR1	EML2	COL5A1	MAST4	FURIN	CPXM1
EPS15	CCR5	ENTPD1	COL9A2	MYO5A	GCNT1	CRADD
ERI1	CD2	ERI1	COMMD7	NAMPT	GRN	CREB3L2
ICOS	CD63	ETV7	CRADD	NCOA3	HIF1A	CSF1
IFI6	CD7	EVA1B	CREB3L2	NETO2	HIVEP3	CTSC
IL1R2	CD74	FBXO45	CST7	PDGFA	ICOS	DPYSL2
IL2RA	CD79B	FKBP1A	CTNNA1	PHACTR2	IKZF4	DYNC1I2
IL2RB	CD80	FNDC3B	CTSC	PHTF2	IL1R2	EGR3
IL2RG	CD82	FUCA2	CXCR6	PTP4A3	IL1RL1	ENTPD1
ISG15	CD83	GADD45A	DPYSL2	SAT1	IRAK2	ETV7
LGALS1	CDIP1	GCNT1	ENO1	SEC14L1	IRF5	FAM126A
LGALS3	CDKN1A	HAP1	ENTPD1	SGMS1	ISG15	FNDC3B
LY6E	CDKN2A	HAVCR2	ERI1	SLAMF1	LAMP2	FUCA2
MAPKAPK3	CEACAM1	HIVEP3	FBLN7	SLC16A1	LAPTM4B	GALM
MRPS6	CLIC1	ICOS	GALM	SOX4	MAP2K3	GCNT1
MX1	CMTM6	IGFLR1	GAPDH	TNFRSF13B	MAPKAPK2	GEM
MYL6	COL9A2	IKZF4	GLA	TNFRSF1B	MAPKAPK3	HAVCR2
NAMPT	CREB3L2	IL1R2	GRAMD4	TPP1	MRPS6	HIVEP3
NCF4	CREM	IL1RL1	HLA-DRB4	WLS	MVP	HMOX1
NDUFV2	CSF1	IL2RA	HSP90AA1	ZBTB32	MXD1	HSPA1A
NFKBIZ	CST7	IL2RB	IFI27		NAMPT	ICOS
OAS1	CTLA4	KAT2B	IFI35		NCF4	IFT27
PELI1	CTNNA1	KLHDC7B	IL18R1		NDRG1	IKZF4
PGK1	CTSC	LAPTM4B	IL1R1		NINJ1	IL1A
PHTF2	CTSD	LAX1	IL1R2		OSBPL3	IL1R1
PLP2	CXCR3	LAYN	IRF4		PICALM	IL1R2
PRDM1	CXCR6	LEPROT	ITGB2		PLP2	IL1RL1
PRDX3	DDIT4	LTA	KAT2B		PMAIP1	IL1RN
PRNP	DDX24	MAP1LC3A	LAPTM4B		RHBDD2	IL2RA
PTTG1	DPYSL2	MAST4	LAX1		RHOC	IL4R
RPS27L	DUSP2	MGST2	LAYN		SAMSN1	IRAK2
S100A4	DUSP4	MICAL2	LGALS1		SDC4	IRF5
S100A6	DYNLL1	MINPP1	LTA		SEC14L1	KSR1

Table S6. Overlap of tumor-infiltrating Treg signatures with significantly upregulated genes in uTregs vs bTregs (% of overlapping genes/genes in signature).

Overlap between uTregs and tumor-infiltrating Treg signatures						
Tirosh et al	Zheng et al	De Simone et al.	Pacella et al.	Toker et al.	Magnuson et al.	Plitas et al.
SAMSN1	EDARADD	MREG	MAPK6		SKAP2	LAPTM4B
SAT1	ENO1	NAB1	MFHAS1		SNX9	LAYN
SDC4	ENTPD1	NCF4	MGST2		SSH1	MAP2K3
SDF4	ERI1	NDFIP2	MYO5A		TMBIM1	MGST2
SLAMF1	ETV7	NETO2	MZB1		TNFRSF18	MICAL2
SNX5	FAM129A	NUSAP1	NAB1		TNFRSF1B	MIR155HG
SPPL2A	FBLN7	PAM	NFIL3		TNFRSF4	MRPS6
SQSTM1	FKBP1A	PARD6G	PAM		TNFRSF8	MYO1E
TANK	FOS	PDGFA	PMAIP1		TNFRSF9	NAGA
TFRC	FOSL2	PRDX3	PMVK		TNIP2	NCF4
TMED9	FYCO1	PTP4A3	PRDX5		TRAF1	NEDD9
TMEM173	GABARAPL1	PTTG1	PRNP		TRAF3	NFKB2
TNFRSF18	GADD45A	RBKS	PSTPIP1		UEVLD	NR4A1
TNFRSF1B	GADD45G	RDH10	RGS1		VIM	NTRK1
TNFRSF4	GALM	RYBP	RHOB		ZNRF1	PARD6G
TPM4	GAPDH	SECTM1	SAMD9			PARPBP
TPP1	GCNT1	SLC16A1	SCD			PDGFA
TXN	GOLGA8A	SNAP47	SDC4			PDIA6
TYMP	GPI	SOX4	SETBP1			PHLDA1
VDR	GRN	SPATS2L	SFT2D1			PIK3AP1
	GSTO1	SSH1	SH2D1A			PSEN1
	HAVCR2	SYT11	SH2D2A			PTP4A3
	HERPUD1	TFRC	SLC20A1			PTTG1
	HLA-DQB1	THADA	SOCS1			RBBP8
	HLA-DRB1	TMEM184C	SPPL2A			RBKS
	HLA-J	TNFRSF18	TFRC			RHOC
	HNRNPLL	TNFRSF4	TMCO1			SDC4
	HSPA1A	TNFRSF8	TMEM109			SETBP1
	HSPB1	TNFRSF9	TNFRSF1B			SH2D2A
	ICOS	TOX2	TNS3			SLAMF1
	IFI6	TPP1	TPI1			SLC4A2
	IGFLR1	TRAF3	TTYH3			SLCO4A1
	IKZF4	TSPAN17	ZBED2			SNX5
	IL10RB	VDR	ZBTB32			SPATS2L
	IL18R1	YIPF6	ZFAND5			THADA
	IL1R1	ZBED2	ZFP36L1			TNFRSF18
	IL1R2	ZNF282				TNFRSF1B
	IL2RA					TNFRSF4
	IL2RB					TNFRSF8
	IL4R					TNFRSF9
	IQGAP1					TNIP2
	IRF5					TNIP3
	ISG15					TNS3
	IVNS1ABP					TRAF1
	KAT2B					TYMP
	KDM5B					UBASH3B
	KIF20B					VDR
	LAPTM4A					VRK2
	LAPTM4B					XXYLT1
	LAT2					ZBED2
	LAYN					
	LDHA					
	LEPROT					
	LGALS1					
	LGALS3					
	LINC00963					
	LRPAP1					

Table S6. Overlap of tumor-infiltrating Treg signatures with significantly upregulated genes in uTregs vs bTregs (% of overlapping genes/genes in signature).

Overlap between uTregs and tumor-infiltrating Treg signatures						
Tirosh et al	Zheng et al	De Simone et al.	Pacella et al.	Toker et al.	Magnuson et al.	Plitas et al.
	LTA					
	LYST					
	MAF					
	MAP2K3					
	MAPKAPK3					
	MAST4					
	MCL1					
	MICAL2					
	MIR497HG					
	MKNK1					
	MRPS6					
	MYO5A					
	MZB1					
	NAMPT					
	NCF4					
	NCOA3					
	NDFIP2					
	NEDD9					
	NRBP1					
	OAS1					
	P2RY10					
	PAM					
	PDCD1					
	PDIA6					
	PELI1					
	PFKFB3					
	PGK1					
	PHACTR2					
	PHLDA1					
	PHPT1					
	PHTF2					
	PIM3					
	PKM					
	PLTP					
	PMAIP1					
	PMF1					
	PMF1-BGLAP					
	PPP1CB					
	PRDM1					
	PRDX1					
	PRDX3					
	PRDX5					
	PRF1					
	PRKAR1A					
	PRNP					
	PTP4A1					
	PTP4A3					
	PTPN22					
	PTPN7					
	PTTG1					
	RAB10					
	RAB11FIP1					
	RAB8B					
	RALGDS					
	RBPJ					
	RGS1					
	RHBDD2					

Table S6. Overlap of tumor-infiltrating Treg signatures with significantly upregulated genes in uTregs vs bTregs (% of overlapping genes/genes in signature).

Overlap between uTregs and tumor-infiltrating Treg signatures						
Tirosh et al	Zheng et al	De Simone et al.	Pacella et al.	Toker et al.	Magnuson et al.	Plitas et al.
	RHOC					
	RNF19A					
	RPS27L					
	SAMSN1					
	SAT1					
	SCO2					
	SDC4					
	SDF4					
	SERPINE2					
	SFT2D1					
	SH2D2A					
	SKAP2					
	SLA					
	SLAMF1					
	SLC16A1					
	SLC3A2					
	SLC5A3					
	SNX9					
	SPATS2L					
	SPPL2A					
	SQSTM1					
	SRA1					
	SRGN					
	STAT3					
	SURF4					
	SYT11					
	TANK					
	TFRC					
	THADA					
	TMCO1					
	TMEM173					
	TMEM50A					
	TMX1					
	TNFAIP3					
	TNFRSF13B					
	TNFRSF18					
	TNFRSF1B					
	TNFRSF4					
	TNFRSF9					
	TNIP3					
	TOX2					
	TPI1					
	TPM4					
	TPP1					
	TRAF1					
	TRAF3					
	TRPS1					
	TSPAN13					
	TSPYL2					
	TYMP					
	UBASH3B					
	VDR					
	VMP1					
	ZBED2					
	ZBTB32					
	ZFP36L1					

Table S7. Genes most often shared between 7 tumor-infiltrating Treg signatures

Name	*# Signatures shared*	*DE ^{pb}uTreg vs bTreg?*	*DE ^{pb}uTreg vs ^{inc}Treg?*	*In uTreg core?*
IL1R2	7	Yes	No	Yes
TNFRSF1B	6	Yes	Yes	Yes
CTSC	6	Yes	Yes	Yes
LAPTM4B	6	Yes	No	Yes
DPYSL2	6	Yes	No	Yes
CREB3L2	6	Yes	No	No
ICOS	6	Yes	No	No
CCR8	6	No	No	No
CSF2RB	6	No	No	No
GLRX	6	No	No	No
MAGEH1	6	No	No	No
ENTPD1	5	Yes	Yes	Yes
IL2RA	5	Yes	Yes	Yes
NCF4	5	Yes	Yes	Yes
SDC4	5	Yes	Yes	Yes
TNFRSF4	5	Yes	Yes	Yes
CRADD	5	Yes	No	Yes
LAYN	5	Yes	No	Yes
TNFRSF18	5	Yes	No	Yes
BCL2L1	5	Yes	Yes	No
GCNT1	5	Yes	No	No
TIGIT	5	No	Yes	No
EBI3	5	No	No	No
F5	5	No	No	No
FOXP3	5	No	No	No
GBP2	5	No	No	No
IKZF2	5	No	No	No
IL12RB2	5	No	No	No
TBC1D8	5	No	No	No
ACP5	4	Yes	Yes	Yes
BATF	4	Yes	Yes	Yes
ERI1	4	Yes	Yes	Yes
NAMPT	4	Yes	Yes	Yes
PTTG1	4	Yes	Yes	Yes
TFRC	4	Yes	Yes	Yes
TPP1	4	Yes	Yes	Yes
VDR	4	Yes	Yes	Yes
CD80	4	Yes	No	Yes
COL9A2	4	Yes	No	Yes
IKZF4	4	Yes	No	Yes
IL1R1	4	Yes	No	Yes
KAT2B	4	Yes	No	Yes
LTA	4	Yes	No	Yes
PTP4A3	4	Yes	No	Yes
TNFRSF9	4	Yes	No	Yes
ZBED2	4	Yes	No	Yes
MRPS6	4	Yes	Yes	No
SLAMF1	4	Yes	Yes	No
ARHGEF12	4	Yes	No	No
CCND2	4	Yes	No	No
CD7	4	Yes	No	No
CD79B	4	Yes	No	No
HTATIP2	4	No	Yes	No
CARD16	4	No	Yes	No
SIRPG	4	No	Yes	No
DFNB31	4	No	No	No
DUSP16	4	No	No	No
FCRL3	4	No	No	No
FLVCR2	4	No	No	No
GBP5	4	No	No	No
MYO5C	4	No	No	No
SYNGR2	4	No	No	No
TMPRSS6	4	No	No	No
UGP2	4	No	No	No
